# Heterotrimeric G–proteins in *Picea abies* and their regulation in response to *Heterobasidion annosum s.l.* infection

**DOI:** 10.1186/s12870-015-0676-1

**Published:** 2015-12-12

**Authors:** Sophie de Vries, Miguel Nemesio-Gorriz, Peter B. Blair, Magnus Karlsson, M. Shahid Mukhtar, Malin Elfstrand

**Affiliations:** Department of Forest Mycology and Plant Pathology, Uppsala Biocenter, Swedish University of Agricultural Sciences, Uppsala, Sweden; Institute of Population Genetics, Heinrich Heine-University, Düsseldorf, Germany; Department of Biology, The University of Alabama at Birmingham, Birmingham, AL USA

**Keywords:** *Picea abies*, Heterotrimeric G-protein, Gγ-subunit, Evolution, *Heterobasidion annosum*

## Abstract

**Background:**

Heterotrimeric G-proteins are important signalling switches, present in all eukaryotic kingdoms. In plants they regulate several developmental functions and play an important role in plant-microbe interactions. The current knowledge on plant G-proteins is mostly based on model angiosperms and little is known about the G-protein repertoire and function in other lineages. In this study we investigate the heterotrimeric G-protein subunit repertoire in Pinaceae, including phylogenetic relationships, radiation and sequence diversity levels in relation to other plant linages. We also investigate functional diversification of the G-protein complex in *Picea abies* by analysing transcriptional regulation of the G-protein subunits in different tissues and in response to pathogen infection.

**Results:**

A full repertoire of G-protein subunits in several conifer species were identified *in silico*. The full-length *P. abies* coding regions of one Gα-, one Gβ- and four Gγ-subunits were cloned and sequenced. The phylogenetic analysis of the Gγ-subunits showed that PaGG1 clustered with A-type-like subunits, PaGG3 and PaGG4 clustered with C-type-like subunits, while PaGG2 and its orthologs represented a novel conifer-specific putative Gγ-subunit type. Gene expression analyses by quantitative PCR of *P. abies* G-protein subunits showed specific up-regulation of the Gα-subunit gene *PaGPA1* and the Gγ-subunit gene *PaGG1* in response to *Heterobasidion annosum sensu lato* infection.

**Conclusions:**

Conifers possess a full repertoire of G-protein subunits. The differential regulation of *PaGPA1* and *PaGG1* indicates that the heterotrimeric G-protein complex represents a critical linchpin in *Heterobasidion annosum s.l.* perception and downstream signaling in *P. abies.*

**Electronic supplementary material:**

The online version of this article (doi:10.1186/s12870-015-0676-1) contains supplementary material, which is available to authorized users.

## Background

Heterotrimeric G-proteins are protein complexes consisting of three subunits (α-, β- and γ-subunit). They are present throughout the plant, animal and fungal kingdoms. Having the ability to recognize and respond to various internal and external stimuli, they regulate many different developmental and environmental responses, such as cell proliferation, cell wall composition, various hormone responses, ion channel regulation, stomatal opening and closure, sugar signaling, pathogen and elicitor responses [1–12].

In contrast to the classical model of G-protein activation, known from fungi and animals, many plants show a strong self-activation of the complex, possibly resulting from comparably more fluctuant and dynamic helical protein domain motions [9, 13, 14]. A conformational change in the Gα-subunit will release the Gβγ-dimer and by that activate downstream signalling pathways *via* either the Gα-subunit and/or the Gβγ-dimer [15, 16]. Completion of the cycle by inactivation of the heterotrimeric G-protein complex seems to differ not only in plants and animals, but even within the plant kingdom [9].

Downstream signalling of the Gα-subunit as well as the Gβγ-dimer [3] can act both synergistically and antagonistically [15]. Pandey et al. [17] assessed different models for the downstream signal propagation and found that one signalling component can only explain a partial range of the possible reactions, indicating that both parts are involved and needed for the variability in heterotrimeric G-protein signalling and function. Specificity in signalling is partially determined by the mutually exclusive expression patterns of the Gγ-subunits in *Arabidopsis thaliana,* although e.g. subunit specificity in flowering signalling cannot be explained with this hypothesis [18]. Additionally, functional diversity is hypothesised to be determined by the number and sequence variation of the complex components, e.g. in animals and fungi a wide variety of Gα-subunits can account for functional diversity [19, 20]. Plants however, possess a small Gα- and Gβ-inventory [9], implying that functional diversity of the plant heterotrimeric G-protein complex is dependent on the number and variation of Gγ-subunits [21].

Accordingly, Gγ-subunits in plants form a small gene family with up to three members that usually show strong sequence diversification [9, 22]. Phylogenetically, plant Gγ-subunit sequences can be classified into three subtypes [22], based on the sequence, the length of their C-terminal region and the motifs therein. A-type-like Gγ-subunits are short proteins containing a C-terminal CAAX motif similar to fungal and animal Gγ-subunits [22], and are the only Gγ-subunit type identified in green algae [23]. The B-types are also short proteins, but have diverged in monocots and dicots possessing the C-terminal motifs KGSDFS and SRXXKRWI, respectively [22]. Trusov and colleagues [22] found no B-type-like sequence in gymnosperms, prompting them to suggest that the B-type diverged from the A-type after the split of gymnosperms and angiosperms between 300 My ago (mya) to 150 mya (based on Pires and Dolan [24]), with a secondary loss in the Brassicaceae. The C-types are longer proteins with a cysteine-rich C-terminus, but the length varies considerably in this group [22]. Interestingly, the moss *Physcomitrella patens* is predicted to have a Gγ-subunit not represented in spermatophyta [22], suggesting that additional Gγ-subunit types may be discovered.

In line with their important functional roles as switches between signal perception and transduction, transcriptional regulation of heterotrimeric G-proteins towards environmental and developmental cues are studied in detail in angiosperms [21, 25–28], and add further support to sequence variation as a key in the broad variety of signalling functions. Analyses of gene expression patterns in *A. thaliana* reveal omnipresent *AGB1* (Gβ) expression that coincide with the Gγ-subunit *AGG1*- and *AGG2*-expression, although the latter two are expressed tissue dependent and mostly mutually exclusive [21].

Lately, G-protein signalling is established as a major component in pathogen responses in both monocots and dicots. Suharsono et al. [29] showed that in rice, the Gα-subunit is an important intermediary of defence responses activated by *Magnaporthe grisea* elicitors, which suggest a role of the Gα-subunit in effector triggered immunity (ETI). However, several subunits of the heterotrimeric G-protein complex respond to microbe associated molecular patterns (MAMPs) [12, 30, 31], indicating a role in pattern triggered-immunity (PTI). In *A. thaliana*, activation of PTI require functional Gβ- and certain Gγ-subunits, while the only C-type Gγ-subunit, *AGG3*, does not seem to be involved in PTI [30]. This suggests functional differentiation in the G-protein subunit repertoire in *A. thaliana*, as well as a species specific usage of the heterotrimeric G-protein repertoire. In line with this, the heterotrimeric G-protein components are required for host and non-host resistance in *A. thaliana*, with the exception of AGG3 [32]. Lee and colleagues [32] also showed that all involved subunits are significantly higher expressed during biotic stress.

Despite being such an important signalling switch, research on heterotrimeric G-proteins is focussed on annual plants. In plants with perennial life styles, such as trees, abiotic and biotic stress are enduring threats that the plants constantly must react to. A quick and functionally specific switch may thus be crucial for the plants longevity. Also, information on the G-protein subunit repertoire in gymnosperms would add important information on heterotrimeric G-protein evolution. Yet, despite their evolutionary history and their ecological and economic importance, our knowledge on heterotrimeric G-proteins in gymnosperms is very superficial. Mostly, Gα-, Gβ- and Gγ-subunit gene sequences in Pinaceae are predicted based on expressed sequence tag (EST) sequences [9, 22]. This data suggest that Pinaceae, like most angiosperms, possess one Gα-, one Gβ- and a small family of Gγ-subunit genes. However, with the aid of the newly published first genome from the conifers, the Norway spruce [*Picea abies* (L.) Karst.] genome [33], additional information may be gained.

In Europe the economically most important pathogen on Pinaceae is the basidiomycete fungus *Heterobasidion annosum* (Fr.) Bref. *sensu lato* (*s.l.*). It is a necrotrophic pathogen specialized on conifers and its spread parallels that of its host (reviewed by Korhonen and Stenlid, [34]). Independent of the co-evolutionary history, the defense responses triggered by *H. annosum s.l.* in *P. abies* are suggested to be non-specific [35–37], resembling PTI. In Europe, two *Heterobasidion* species are known to infect *P. abies*, *H. annosum sensu stricto* (*s.s*) and *H. parviporum* [38] causing stem and root rot in the infected tree, devaluing the timber and increasing the risk of wind-throw [34].

In this study we used the newly available Norway spruce genome in combination with EST databases to elucidate the heterotrimeric G-protein complex in Pinaceae for evolutionary analyses. Our phylogenies, including more Pinaceae sequences, are coherent with previous studies on plant evolution, with regards to Gα- and Gβ-subunits. The phylogeny of Gγ-subunits indicate lineage-specific radiation. We identify a dicot-specific A-type, as well as a novel gymnosperm type not represented in more basal or higher lineages. Sequence diversifications indicate subfunctionalization of the different Gγ-subunits in the Pinaceae, which is supported by tissue specific expression in Norway spruce. We observe changes in the expression patterns of the heterotrimeric G-protein subunit genes in response to wounding, methyl jasmonate (MeJA), abscisic acid (ABA), a saprotrophic fungus and the necrotrophic pathogen *H. annosum s.l.* This consistent with a pattern-triggered response that is either independent or upstream of the hormone signalling pathways. To the best of our knowledge we present here the first report on heterotrimeric G-protein signalling in perennial species towards biotic stresses.

## Results

### Conifers encode and express a full repertoire of heterotrimeric G-protein subunits

Previous studies [9, 22] had already reported some sequences of the Pinaceae heterotrimeric G-protein complex, based on EST sequences. We identified one Gα-, one Gβ- and four Gγ-subunit-like gene sequences in the *P. abies* genome [33]. The same number was identified in *Picea sitchensis*, while one Gβ- and only three Gγ–subunit-like sequences were found in *Picea glauca* and *Pinus taeda*. The Gα-subunit-like sequences for these two species were reported previously by Urano et al. [9]. In addition, we also identified Gα-subunit-like sequences in additional *Pinus* species. All sequences used in the current study are listed in Additional file 1. While we found gene models for all subunits in the *P. abies* genome assembly, only *PaGG1* had a high confidence gene model that covered the whole sequence. This was not surprising, due to the large genome size, long introns, and high content of repetitive regions, which limited the *P. abies* assembly [33]. We confirmed the *in silico* identified Gα-, Gβ- and Gγ-like genes from *P. abies* by cloning and sequencing the full-length coding sequences from cDNA libraries [KM197161 (*PaGPA1*) and KC825350.1-KC825354.1 (*PaHGB1* – *PaGG4*)]. In our subsequent studies we used the sequences determined from *P. abies* cDNA.

**Fig. 1:**
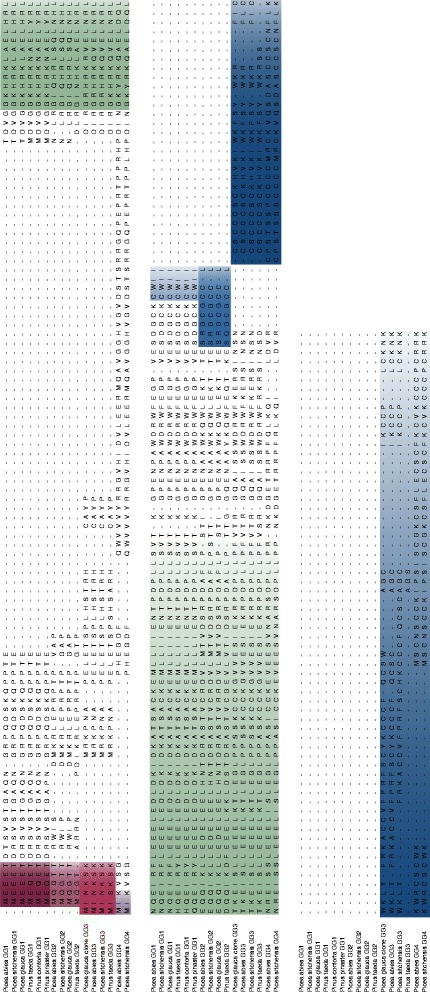
Alignment of the isolated *P. abies* Gγ-subunits. Alignment of the predicted amino acid sequences of PaGG1, PaGG2, PaGG3 and PaGG4. The alignment was done using CLUSTALW in MEGA5.0. The conserved N-terminal motifs of Pinaceae GG1, GG2, GG3 and GG4 are highlighted (in purple). The conserved region in Gγ-subunits found in the plant kingdom is highlighted in green. The type-specific C-termini are highlighted in blue

In general, the G-protein repertoire in Pinaceae was similar in numbers between the investigated species. The lengths of the predicted G-protein-like subunit amino acid sequences were conserved between species in Pinaceae, with the exception of the putative *P. taeda* GG3 that was 38 amino acids shorter than the orthologous PaGG3 (Fig. 1). The predicted molecular weights of the Gα–subunit-like PaGPA1 and the Gβ-subunit-like PaHGB1 proteins from *P. abies* were 45.4 and 41.4 kDa, respectively, while the molecular weights of the Gγ-subunit-like proteins were predicted to be equal to, or lower than 23.4 kDa (Table 1).

**Table 1:** Molecular data of the predicted *P. abies* G-protein subunits

	Predicted	Gene			Predicted	Amino acid motifs		
Gene	transcript	model	NCBI accession	ORF (bp)	MW (kDa)	N-terminal	C-terminal^a^	Type^a^
*PaGPA1*	comp91545_c0_seq2	MA_95177	KM197161.1	1016	45.4	-	-	-
	/comp92545_c1_seq1	/MA_9999470g0010						
*PaHGB1*	comp75963_c0_seq1	MA_10429560g0010	KC825350.1	1134	41.4	-	-	-
*PaGG1*	comp86733_c0_seq1	MA_87554g0010 /MA_183273g0020	KC825351.1	336	12.5	MEEET	CaaX	A
*PaGG2*	comp85221_c0_seq1	MA_202946g0010	KC825352.1	318	12.0	MQGT	SRGCGCCL	A-like
*PaGG3*	comp79582_c0_seq1	MA_173928g0010	KC825353.1	513	19.8	MINKS	C-rich	C
*PaGG4*	comp42525_c0_seq1	MA_66599g0010	KC825354.1	624	23.4	MIK	C-rich	C

^a^Predicted transcript and gene models in the *P. abies* 1.0 gene catalog (http://congenie.org/)^b^Definitions according to [22]

The predicted Gγ-subunits separated into two short (PaGG1 = 336 amino acids and PaGG2 = 318 amino acids) and two long (PaGG3 = 513 amino acids and PaGG4 = 624 amino acids) proteins (Table 1). The Gγ-like subunits were divided into four different types, based on the highly variable N- and C-terminal parts. We identified four conserved N-terminal motifs for the different Gγ-subunit-like proteins in Pinaceae: GG1 - MEEET (*Picea*)/ MEQET (*Pinus*), GG2 - MQGT (*Picea/Pinus*), GG3 - MINKS (*Picea*)/ MISKS (*Pinus*) and GG4 - MIK (*Picea*) (Fig. 1). Further, they showed specific C-termini (Fig. 1): PaGG1 contained a CAAX motif (CWII) that classified PaGG1 and its orthologs as an A-type Gγ-subunit; PaGG3 and PaGG4 had long C-termini with high cysteine contents of 29 % (PaGG3) and 30 % (PaGG4), representing C-type Gγ-subunits; while the short subunit PaGG2 and its orthologs contained a novel motif (SRGCGCCL), previously not shown to be present in monocots or dicots [22]. PaGG1 but not PaGG2 show a complete G-protein γ subunit-like (GGL)-domain [39] (Additional file 2).

### Conifers contain a novel Gγ-subunit type

To better understand how the heterotrimeric G-protein complex has evolved we conducted phylogenetic analyses of the components. Our phylogenetic analysis confirmed that the Gα-subunit-like and Gβ-subunit-like sequences mainly follow previously published plant phylogenies [24, 40, 41] (Additional files 3, 4 and 5).

**Fig. 2:**
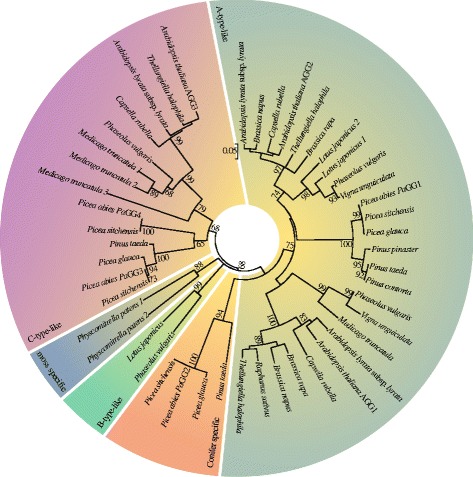
Evolutionary relationships of the Gγ-subunits in the plant kingdom. Neighbor-Joining tree of the Gγ– subunit-like sequences of Pinaceae, Fabaceae, Brassicaceae and the moss *Physcomitrella patens*; A-type-like sequences containing a CAAX-box motif are highlighted in olive, B-type-like sequences are highlighted in green, C-type-like sequences having long cysteine-rich C-termini are highlighted in pink, conifer specific short sequences are highlighted in orange and moss sequences in blue . Bootstrap support over 65 is associated with lineages

The resulting phylogenetic tree for Gγ-subunit-like sequences demonstrated type-dependent, rather than plant evolution dictated topology (Fig. 2). We obtained clusters representing A-type-like, B-type-like and C-type-like proteins, respectively (Fig. 2, Additional file 6). PaGG1 and its orthologs in Pinaceae clustered with the angiosperm A-type-like sequences (Fig. 2). In agreement with the unique C-terminal ending, PaGG2 and its conifer homologs formed a separate clade, related with the A-type-like cluster (Fig. 2). This is in accordance with the higher amino acid similarity between PaGG1 and PaGG2 (56.5 %), compared to the overall mean similarity between all *P. abies* Gγ-subunit-like types (29.9 %). The C-type-like cluster was split into two groups: one containing dicot and the other conifer proteins, including PaGG3 and PaGG4 (Fig. 2).

### Yeast two hybrid assay with conifer G-protein subunits

**Fig. 3:**
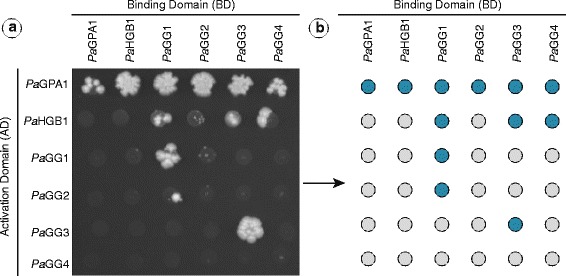
Interactions of P. abies G-protein subunits. Yeast-2-Hybrid screening of direct interactions of the identified P. abies G-protein subunits on –LTH agar plates (**a**). The experimental matrix of G-Protein AD/DB mates: blue indicates interaction on -LTH agar plates (**b**)

For heterotrimeric G-proteins to be functional, the Gα-, Gβ- and Gγ-subunits must physically interact with each other as it has been shown in other model organisms [12, 42]. To analyse this protein-protein interactions among the members of G-proteins in Norway spruce, we performed a comprehensive yeast two-hybrid assay. All subunits were fused with both a GAL4 activator domain (AD) and a GAL4 binding domain (DB) individually. These constructs were subsequently transformed into haploid yeast strains and mated in a simple crosswise matrix (Fig. 3). Protein-protein interactions were scored based on yeast growth on selection media but no growth on the autoactivation media. As expected PaGPA1-AD interacted with PaHGB1-DB (Fig. 3). Also, PaHGB1-AD showed interaction with the Gγ-subunits PaGG1-DB, PaGG3-DB and PaGG4-DB, but not with PaGG2-DB (Fig. 3). Instead, PaGG2-AD interacted with PaGG1-DB but not with itself, PaGG3-DB or PaGG4-DB.

As a limited interaction between Gγ- and Gα-subunits have been reported [42] in the absence of the Gβ-subunit in mammalian systems [43–45] we also tested the interaction between PaGPA1 and the identified Gγ-subunits. Indeed, the PaGPA1-AD interacted with PaGG1-, PaGG2-, PaGG3- and PaGG4-DB (Fig. 3).

### Different levels of sequence diversification indicate subfunctionalization of Gγ-subunits

**Fig. 4:**
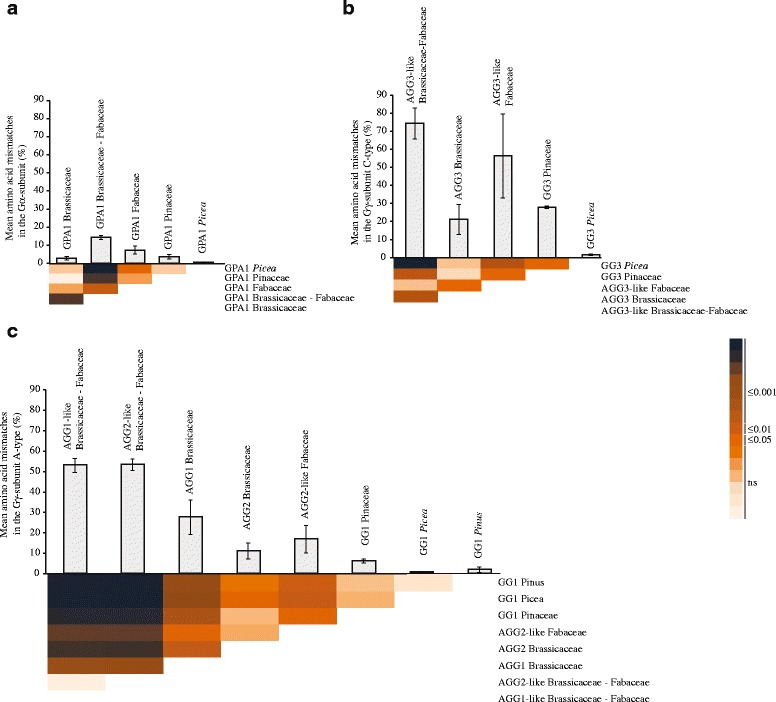
Conservation of amino acid sequences of Gα and Gγ in Pinaceae and selected angiosperm families. Conservation level of GPA1-like protein sequences in Fabaceae, Brassicaceae, Pinaceae and between the Fabaceae and Brassicaceae (**a**). Conservation level of long Gγ-subunit/C-type-like protein sequences in Fabaceae, Brassicaceae, Pinaceae and between the Fabaceae and Brassicaceae (**b**) and amino acid conservation level of short Gγ-subunit-like protein sequences in Fabaceae, Brassicaceae, Pinaceae*, Picea, Pinus* and between the Fabaceae and Brassicaceae (**c**). The bar diagram shows the mean amino acid mismatches per protein sequence length in percentage of the Gα-and Gγ-subunits of the Pinaceae, Fabaceae and Brassicaceae estimated in a pairwise sequence comparison within the on the x-axis indicated sequence clusters. Error bars indicate the standard deviation. The heatmap gives the significant differences estimated using one-way ANOVA and the post-hoc Tukey-test. The color scale corresponds to the significance levels and is applied to all heatmaps

Gγ-subunits show a low overall conservation in the plant kingdom, which suggests that sequence variation may result in functional divergence. We therefore assessed if Gγ-subunits evolve under different evolutionary constraints, by performing pairwise comparisons of amino acid conservation in the A- and C-type Gγ-subunit clusters in Pinaceae, Brassicaceae and Fabaceae. Brassicaceae and Fabaceae were chosen as valid angiosperm comparisons as their divergence times (125 mya [46]) are similar to the divergence time between the genera *Picea* and *Pinus* (145 mya [40, 41]). For comparison, we also conducted this analysis for the Gα-subunit in the same taxa. The lowest sequence variation was detected for the Gα-subunit (Fig. 4a). The highest sequence variation was found in angiosperm C-type-like Gγ-subunits (Fig. 4b). Sequence variation was significantly (*P* ≤ 0.05) lower for all conifer Gγ-subunit-like and the Gα-subunit-like sequences, compared with their angiosperm equivalents (Fig. 4).

### Differential Gγ-subunit gene expression indicate subfunctionalization in *P. abies*

**Fig. 5:**
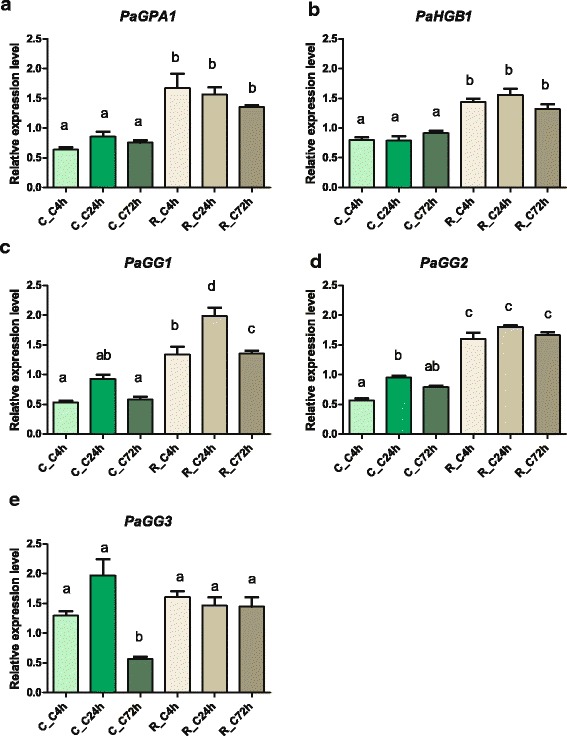
Tissue specificity of G-protein subunits in *P. abies* . The relative expression values in cotyledons and roots of *P.abies* seedlings of *PaGPA1* (**a**); *PaHGB1* (**b**); *PaGG1* (**c**); *PaGG2* (**d**) and *PaGG3* (**e**) The relative expression in cotelydons (C) and roots (R) at 4-, 24- and 72 relative to time point t0 = 0 h is indicated is shown. Numbers in the sample code represent the time points at which the tissues were collected. The letters on the bars indicate different statistical groups and the standard deviation is given by error bars (N = 3)

The observed differences in amino acid conservation between the Gγ-subunit types may suggest sub- or neofunctionalization. To test this, we studied gene expression of *PaGPA1, PaHGB1, PaGG1, PaGG2* and *PaGG3* in cotyledons and roots of *P. abies* seedlings at 4, 24 and 72 h after transfer to fresh medium (Fig. 5). Roots showed a higher expression (*P* ≤ 0.05) of *PaGPA1, PaHGB1, PaGG1* and *PaGG2* compared to cotyledons over time. *PaGG3* showed stable expression levels over time and tissues, although with decreased levels in cotyledons after 72 h.

After having established basal expression levels, we investigated the effect of abiotic and biotic stress on G-protein subunit gene expression. Expression of *PaGPA1, PaHGB1, PaGG1, PaGG2* and *PaGG3* was analysed in cotelydons and roots at 4, 24 and 72 h post infection (hpi) with *H. annosum s.s.* conidiospores. In a separate experiment, seedlings were treated with the defense signalling hormones abscisic acid (ABA) and methyl jasmonate (MeJA), as well as a wounding treatment. Expression levels of *PaGPA1*, *PaHGB1*, *PaGG1* and *PaGG3* were significantly (*P* ≤ 0.05) induced in *P. abies* roots after 72 hpi with *H. annosum s. s.* (Table 2). The induction of *PaGPA1* was detectable already at 24 hpi, and reached a five-fold induction at 72 hpi. Expression of *PaGG3* was induced in cotyledons, as well as in roots. Hormonal treatments or wounding did not induce any changes in expression of any gene (Additional file 7).

**Table 2:** Transcriptional regulation of G-protein subunits seedling roots in response to *H. annosum s.s*

	Cotelydons		Roots	
	24 hpi^a^	72 hpi^a^	24 hpi^a^	72 hpi^a^
*PaGPA1*	1.2 ± 0.0	1.4 ± 0.1	2.9 ± 0.8**	4.9 ± 3.0*
*PaHGB1*	1.3 ± 0.0	1.0 ± 0.1	1.7 ± 0.1	2.5 ± 1.5*
*PaGG1*	1.2 ± 0.1	0.9 ± 0.1	1.5 ± 0.0	2.8 ± 1.3*
*PaGG2*	1.1 ± 0.1	0.9 ± 0.1	1.2 ± 0.1	2.2 ± 0.9
*PaGG3*	1.1 ± 0.1	2.4 ± 0.5**	1.9 ± 0.0	2.7 ± 0.9*

^a^ Relative expression values of *PaGG1, PaGG2, PaGG3, PaHGB1* and *PaGPA1* in cotelydons and roots of *P. abies* at 24 and 72 h post inoculation (hpi) with *Heterobasidion annosum s. s.* conidia suspension relative to time point t0 = 0 h (N = 3).

* Indicate significantly induced expression compared to the control at *P <*0.05 and >0.01 ** Indicate significantly induced expression compared to the control at *P* <0.01 and > 0.001

**Fig. 6:**
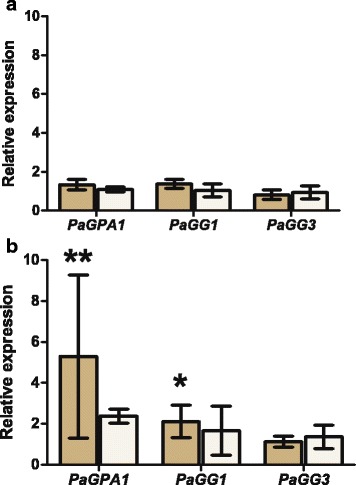
Transcriptional regulation of G-protein subunits in response to *H. parviporum*. Relative expression values of *PaGG1, PaGG3* and *PaGPA1* in bark of 4-year old *P. abies* seedlings inoculated with *H. parviporum* (tan) and *P. gigantea* (open) in relation to wounding 72 h after treatment at the site of wounding and inoculation (**a**) and distal to the inoculation site (**b**). * corresponds to *P <*0.05 and >0.01, ** corresponds to *P* <0.01 and > 0.001

To further investigate the response of *PaGPA1*, *PaGG1*, *PaGG2* and *PaGG3* to *H. annosum s.l.*, their expression was analysed in bark of 4-year old *P. abies* plants subjected to wounding or inoculation with *H. parviporum* or the saprotrophic fungus *Phlebiopsis gigantea*, unable to colonize *P. abies* bark tissue [47]. Expression levels were quantified 72 hpi/wounding directly at the inoculation/wounding site and at a distal position, 2 cm away from the inoculation site. *PaGG2* expression in bark was below the detection limit of the assay. None of the other subunit genes were differentially expressed in response to either fungal inoculum in comparison to wounding at the local site (Fig. 6a). However, at the distal location, expression of *PaGPA1* and *PaGG1* were induced by *H. parviporum* infection, but not by *P. gigantea*, when compared to the wounding control (Fig. 6b).

## Discussion

### Conifers possess a unique short Gγ-subunit type not present in other land plants

In this study we set out to investigate presence and functionality of heterotrimeric G-proteins in woody plants. We focus on the conifer *P. abies* and several of its close relatives. We identified and verified the presence of one Gα-, one Gβ- and four different Gγ-subunit genes in *P. abies* and found the orthologous genes in other conifers. Our survey identified an additional Gγ-subunit gene in *P. abies* and *P. sitchensis*, not present in *P. glauca* and *P. taeda*. The observations for *P. taeda* and *P. glauca* are in accordance with the three Gγ-subunit genes previously reported from Pinaceae [9], and could suggest that the *Picea* lineage gained a fourth Gγ-subunit gene that was later lost in *P. glauca*. However, as the conifer sequences, except *P. abies*, are retrieved from EST databases, we cannot exclude the existance of additional genes.

The four different predicted Gγ-subunit-like protein sequences from *P. abies* can be divided into short and long variants. The modular structures classify PaGG1 as an A-type Gγ-subunit, and PaGG3 and PaGG4 as members of the C-type-like Gγ-subunit group, according to the description by Trusov et al. [22]. We found this to be in complete agreement with their phylogenetic placements in our current study. The phylogeny indicates that GG3 and GG4 are recent duplicates that arose during conifer evolution. Based on our data, the most parsimonious hypothesis indicates the duplication event took place after the split of the genera *Picea* and *Pinus,* with GG3 being the ancestral sequence. The sequence of PaGG2 and its coniferous orthologs contain a novel C-terminal motif matching neither A- or C-type-like sequences, nor the monocot or dicot specific B-type sequences. The phylogenetic analysis, together with the observed high similarity between PaGG2 and PaGG1, suggest that PaGG2 and its orthologs have diverged from the A-type-like clade. Thus, PaGG2 and its orthologs may represent a novel, conifer-specific Gγ-subunit type.

### Conifer Gγ-subunits interact differently with PaHGB1 and PaGPA1

As expected with a single Gα- and Gβ gene PaGPA1 interacted with PaHGB1 in the yeast-2-hybrid screen. The smaller Gγ-subunits are essentially buried in the Gβ-subunit, except for the N-terminus of the Gγ-subunit, [42] forming the Gβγ-dimer [15]; we found that PaHGB1 interacts with the Gγ-subunits PaGG1, PaGG3 and PaGG4 but not with the novel, conifer specific, Gγ-subunit PaGG2; raising a question about PaGG2′s functionality. An inspection of the predicted secondary structure of the PaGG2 protein indicates that PaGG2 forms only one α-helix instead of two in the GGL-domain [39]. Such an incomplete GGL domain may interact only weakly with the Gβ-subunit.

In accordance with previous reports from mammalian systems we found that PaGPA1 also interacted with each one of the *P. abies* Gγ-subunits, including PaGG2. The interactions between mammalian Gγ- and Gα-subunits in the absence of the Gβ-subunit [42–45] have been suggested to depend on the N-terminal region of Gγ proteins [45] protruding from the Gβγ-dimer, and to have a potential effect on the activation of Gα subunits [48] however the corresponding results have not yet been reported from plants.

### Sequence divergence of the heterotrimeric G-protein complex differs between conifers and angiosperms

In most plant species the Gγ-subunits are the only part the heterotrimeric G-protein complex that have more than one gene family member [9]. In addition, they are highly variable in sequence and the differences in their transcriptional responses are suggested as critical factors in the broad role of G-protein responses [18, 21, 22, 32]. High sequence divergence and specific gene regulation are indicators for sub- and/or neofunctionalization. The Gγ-subunit sequences demonstrate a much stronger sequence diversification, especially among C-type-like sequences (≤75 % amino acid substitutions) compared to the Gα-subunits (≤15 % amino acid substitutions). This result is aligned with the variable number of Gγ-subunit genes in most plants [9]. Interestingly, we also show that G-protein subunit sequences in Pinaceae are more conserved compared to their dicot counterparts, irrespectively of subunit type. Knowing that gymnosperms generally present a slower evolution than angiosperms, probably due to their long life-spans and large effective population sizes [49], we attribute this observation to the coniferous lifestyle. Such differences in sequence divergence may indicate functional divergence, which is demonstrated by the significant difference between the Brassicaceae AGG1 and AGG2 orthologue groups that have mutually exclusive gene expression patterns [21].

### The conifer G-protein complex shows specific regulation

The different levels of sequence conservation prompted us to study gene expression of the heterotrimeric G-protein complex in *P. abies* within different tissues. In contrast to the green algae *Chara braunii* [23], we found a ubiquitous but tissue-differentiated expression pattern of all subunits. In this respect, the expression pattern is more similar to what is seen in angiosperms compared to more basal lineages, resembling those reported for the putative orthologs in *Brassica napus* and *A. thaliana* [3, 21, 26, 27]. The *PaHGB1* expression also coincides with expression of *PaGG1* and *PaGG2* as expected for interacting Gβ- and Gγ-subunits, despite that we could not demonstrate an interaction between *PaHGB1* and *PaGG2* in the yeast-two-hybrid assay. Interestingly, the constitutive expression of *PaGG3* in *P. abies* seedlings is in accordance with the constitutive expression of *AGG3* in *A. thaliana* seedlings [3].

### *H. annosum s.l.* triggers G-protein expression in a MAMP-responsive manner

Our interest in functional divergence of the heterotrimeric G-protein responses in pathogen defense signalling led us to study expression patterns of the *P. abies* G-protein subunit genes within different tissues and under different pathogen associated treatments. In the *P. abies-H. annosum s.l.* pathosystem, wounding and pathogen inoculation show a qualitatively similar response, although the response to the pathogen has a higher amplitude and duration [35, 36, 47]. This indicates that the defence responses against *H. annosum s.l.* are MAMP-triggered, but similar to a DAMP-triggered [50] wound response [35–37, 47]. The response also involves hormone triggered defense pathways as JA mediated resistance [35, 47]. Interestingly, the *P. abies* G-protein subunits *PaGPA1, PaHGB1, PaGG3* and *PaGG1* in seedling roots respond to *H. annosum s.s.* treatment, but not to wounding of the seedling. The response in bark of four-year old seedlings was similar, but do not differ between treatments of *H. annosum s. l.* and *P. gigantea,* a non-pathogenic fungus [47] at the treatment site, indicating MAMP-based signalling cues irrespective of seedling age. The differential regulation of *PaGG1* and *PaGG2* in roots of young seedlings suggests functional differentiation between them*,* in accordance with the different levels of sequence conservation between orthologs.

We also observed that wounding responses and the response to the saprotroph *P. gigantea* in inoculated bark on branches of four-year old seedlings will weaken with distance [47], while the response to *H. parviporum* persists. We suggest that this phenomenon occurs because of the colonization of the living bark by the pathogen and the continuous release of MAMPs. Consequently, *PaGPA1* and *PaGG1* are significantly induced at the distal location only in *H. parviporum* treatments. The observation that the response to *H. parviporum* and *P. gigantea* differ agrees with results from Schwacke and Hager’s [51], showing that the amplitude of the *P. abies* response increase with elicitors from *H. annosum s. l.* compared to elicitors from ectomycorrhizal fungi. Based on pharmacological studies the responses observed by Schwacke and Hager [51] have been suggested to be mediated by either an (auto) phosphorylation of a membrane-bound receptor kinase prior to the activation of a G-protein or (and) immediately downstream of the activated G-protein [52]. These observations are in agreement with our results and our suggestion of heterotrimeric G-proteins acting upstream of JA-signaling, even if the specificity of the Gα- subunit activator mastoparan, used in [52], has been questioned [53], it is an interesting observation and we think that it merits further studies the role of PaGPA1 and its orthologs in MAMP perception in Pinaceae.

## Conclusions

*P. abies* possess a full repertoire of G-protein subunits, including a novel conifer-specific short Gγ-subunit type (*Pa*GG2 and its orthologs). However, the functionality of *Pa*GG2 is questionable, given that the protein appears not to interact with *Pa*HGB1. Sequence divergence suggests relaxed evolution of the Gγ-subunits compared to the Gα-subunits, a pattern typical for duplicated genes. Different evolutionary constraints between the Gγ-subunits are concomitant with the different expressional responses towards unchallenged and challenged situtations. This indicates subfunctionalization of the paralogous Gγ-repertoire. Further, differential regulation of *PaGPA1* and *PaGG1* in response to *H. annosum s.l.* infection indicates that the heterotrimeric G-protein complex represents a critical linchpin in pathogen-perception and downstream signalling responses.

## Methods

### Database searches

We conducted blastx and blastp searches in the NCBI nucleotide, protein and EST databases, the Gene Index Project (The Gene Index Databases-Dana Faber Cancer Institute; [54–56], Uniprot (The Uniprot Consortium, 2012), The *P. abies* genome v 1.0 [33] and Phytozome v9.1 [57] to collect our dataset. Our database search was performed in two steps: 1.) GPA1, AGB1, AGG1, AGG2 and AGG3 protein sequences (from *A. thaliana*) were used as the input data to retrieve the first set of sequences and 2.) The validated sequences of this first set were then used to repeat the database search to ensure high coverage of our dataset. The recovered nucleotide sequences were translated into amino acid sequences using the translate function (with standard genetic code) of the Sequence Manipulation Suite [58]. To verify the retrieved dataset we queried The Arabidopsis Information Resource (https://www.arabidopsis.org/) protein database and analysed the hit with highest similarity. Additionally, we assessed the Gα- and Gβ-subunits alignability with the *A. thaliana* sequences and searched for the conserved domains described by Trusov et al. [22] in the possible Gγ-subunits.

The retrieved sequences were combined with identified dicot Gγ-subunits and the full-length Gα-subunits of *P. glauca* and *P. taeda* from the verified dataset published in Urano et al. [9] (Additional file 1) for phylogenetic analyses. The Gα-subunit-like and Gβ-subunit-like datasets include sequences from species in the Brassicaceae, Fabaceae and Pinaceae and *P. patens* Gβ-subunit-like sequence. We created a Gγ-subunit-like dataset with isequences from the Brassicaceae, Fabaceae, Pinaceae and *P. patens.*

We observed unusual valine-rich C-termini in the *Medicago truncatula* Gγ-subunit C-type-like in our datasets. Analyses of the genomic sequences showed frame shifts in the predicted exon-border (Phytozome v9.1 [57]) in all three sequences: Medtr8g021170.1 showed a one-base frame shift in its last exon, Medtr2g042200.1 had a two-bases frame shift in the second to last exon by of Medtr2g042200.1 and in Medtr4g125190.1 a five-bases elongation in the 5′ end of the second to last exon was corrected to gain cysteine-rich C-termini. For further information on the alignments see Additional files 4, 5 and 6.

### Amplification of *P. abies* G-protein sequences

We cloned the full-length heterotrimeric G-protein subunit coding sequences from *P. abies*. The primers were designed based on the retrieved ESTs and nucleotide sequences from three Pinaceae species: *P. sitchensis*, *P. glauca* and *P. abies*. Primer sequences were listed in Additional file 8.

The *PaGPA1* gene appeared to be split into two different predicted transcripts, comp92545_c0_seq1 and comp92545_c1_seq1 in the *P. abies* 1.0 genome database. Amplification of the predicted 1173 bp full-length transcript was performed in a PCR reaction consisting of 1x Dream-Taq green buffer, 0.25 μM of each of the primers, 0.2 mM dNTPs, 6.25U Dream-Taq Polymerase (Fermentas) and 1 μl of *P. abies* cDNA. Initial denaturation was at 95 °C for 5 min, followed by 35 cycles of: 15 s at 95 °C, 20 s at 58 °C and 120 s at 72 °C and a final elongation step of 3 min at 72 °C.

The *PaHGB1* sequence was amplified from *P. abies* cDNA *via* a two-step PCR using the Advantage® 2 DNA polymerase mix (Clontech Laboratories, Inc.), 1:50 diluted PCR product of the first reaction was used as template for the second reaction to increase the product amount.

Gγ-subunit-like sequences *PaGG1, PaGG2, PaGG3* and *PaGG4* were amplified from 3′- and 5′-SMARTer™ RACE cDNA (Clontech Laboratories, Inc.) libraries of *P. abies* infected with *H. parviporum*, according to the manual’s instructions in a two-step PCR approach (*PaGG1, PaGG2* and *PaGG3*) and a nested PCR approach (*PaGG4*).

The PCR products of *PaGPA1, PaHGB1, PaGG2* and *PaGG3* were extracted from agarose gels with the GenJET™ Gel Extraction kit according to manual, while the PCR products of *PaGG1* and *PaGG4* were directly purified with the GenJet™ PCR-purification kit. The purified PCR products were cloned using TOPO®TA Cloning (Life Technologies) according to instructions and plasmids were sequenced at Macrogen (Amsterdam, Netherlands). Good quality sequences were translated into amino acid sequences using the translate function with standard genetic code of the Sequence manipulation suite [58]. We verified all amino acid sequences as heterotrimeric G-protein complex components in TAIR and NCBI as described previously. Secondary structures of the amino acid sequences were predicted using the PreSSAPro software (http://bioinformatica.isa.cnr.it/PRESSAPRO/).

### Phylogenetic analyses

Phylogenetic relationships of the different subunit types of the heterotrimeric G-protein were analysed with MEGA 5.0 [59]. Phylogenies were constructed for all datasets with the Neighbor-joining algorithm, 1000 bootstrap repetitions, p-distance estimations as a statistical model, uniform substitution rates and a partial sequence cutoff value of 95 %. Gα-subunit-like and Gβ-subunit-like sequences were aligned using CLUSTALW with default options, Gγ-subunit sequences were aligned manually due to their high sequence variability.

### Amino acid sequence characteristics of Gγ repertoire in *Picea abies*

Molecular weight predictions and sequence identity and similarity analyses were performed with the Protein molecular weight function and *ident* and *sim* functions of the Sequence manipulation suite [58]. Sequence similarity predictions were based on the alignment in Fig. 1 and similar amino acids were grouped according to the suggestions in MEGA 5.0 [59], for better comparison of the data.

### Conservation of heterotrimeric G-proteins

We estimated sequence divergence as mean amino acid mismatches /sequence length of pairwise comparisonsfor Gα-subunit-like sequences and for A- and C-type-like Gγ-subunits. Every gap was considered a mismatch. In comparisons including at least one incomplete sequence, only the region covered by both sequences was considered. To gain a better understanding about G-protein evolution in Pinaceae we analysed sequence divergence within the following phylogenetic clusters: i) Fabaceae–Brassicaceae, ii) Fabaceae, iii) Brassicaceae, iv) Pinaceae, v) *Picea* and vi) *Pinus*, if the cluster contained more than three different species. The AGG1-like cluster of the Fabaceae was omitted, because the incompleteness of the *Vigna unguiculata* sequence FF393368.1 biased the results due to the high sequence variability. The statistical differences between the clusters were tested using a one-way ANOVA followed by Tukey post-hoc test.

### Biological material

*H. annosum s.s.* isolate Sä16-4 [60] was cultivated on Hagem medium [60] plates at 25 °C in the dark until the plates were covered with mycelia. Conidia were isolated from the surface with autoclaved water and a Drigalski spatula. The suspension was filtered through glass wool. Conidia concentration was determined using a hemocytometer (Bürker, Scherf Präzision).

Seeds of *P. abies* (S09/120) were surface sterilized with 33 % hydrogen peroxide, one drop Tween20 was added and seeds were gently rotated in the sterilization solution for 15 min followed by 6 washes with autoclaved water. Seeds were covered in water and imbibed over night at 4 °C. The seeds were allowed to germinate on water agar and then transferred onto slanted ¼ Schenk-Hildebrandt medium (pH 5.6; Duchefa Biochemie) with 0.35 % gelrite (Duchefa) until developing the first true needles. Seedlings were incubated in a vertical position at 22 °C under long day conditions.

### Gene expression experimental set-up

*P. abies* seedlings used in the expression studies were i) transferred to Schenk-Hildebrandt medium with 10 μM ABA (stock solution 100 mM ABA in 100 % EtOH; Sigma Aldrich), ii) wounded on their hypocotyl with a needle iii) treated with 3 ml of a *H. annosum s.s.* isolate Sä16-4 conidiospore suspension at 1.5 x 10^6^ ± 31 x 10^5^ (SE) spores/ml and iv) treated with MeJA (Sigma Aldrich). Seedlings treated with MeJA were incubated in a closed chromatography chamber with 75 μl 10 % MeJA per 1 l chamber volume. Samples were taken at 0, 4, 24 and 72 h post treatment. Root and cotyledons were collected separately, frozen with liquid nitrogen and stored at −70 °C until further use. Each treatment and control included three biological replicates with five seedlings per replicate.

Expression analyses in *P. abies* bark were done on branches of four years old plants, from the full-sib family S21H982005 originating from the Swedish breeding programme, inoculated with *P. gigantea* (Rotstop S), *H. parviporum* (Rb175) or wounding as described in Arnerup et al. [47]. Samples from the wounding/inoculation site (0–0.5 cm) and a distal location (1.5–2.5 cm) taken 72 hpi were analysed. Three biological replicates per treatment were used.

### Quantitative PCR

Total RNA extraction was done essentially according to the protocol by Chang et al. [61]. Samples were DNase treated with DNase1 (Sigma Aldrich, USA) according to the manufacturer’s instructions and RNA concentration was determined with the NanoDrop (Spectrophotometer ND 1000, Saven Werner). 300 ng of total RNA was reverse transcribed to cDNA with the iScript™ cDNA Synthesis Kit (BIO-RAD, Sundbyberg, Sweden) according to the manufacturer’s instructions .

Quantitative PCR was performed with the SsoFast™ EvaGreen® Supermix (BIO-RAD) according to the instructions in the manual, using 0.3 μM of each primer. The qPCR were carried out in an iQ5™ Multicolor Real-Time PCR Detection System thermo cycler (Bio-Rad) using a program with a 30 s initial denaturation step at 95 °C, followed by 40 cycles of 5 s denaturation at 95 °C and 10 s at 60 °C. Melt curve analyses were used to validate the amplicon. Relative expression (fold change) was calculated using the 2^-ΔΔCT^ method [62]. One-way ANOVA with the Tukey post-hoc test or the Mann–Whitney *U* test in the GraphPad Prism 5.0 statistical package (GraphPad Inc.) was used to test for statistical differences in expression.

### Yeast two hybrid assay among conifer G-protein subunits

*PaGPA1, PaHGB1, PaGG1, PaGG2, PaGG3* and *PaGG4 cDNA* sequences were amplified with Attb primers (Additional file 8) in a PCR reaction consisting of 1x Dream-Taq green buffer, 0.25 μM of each of the primers, 0.2 mM dNTPs, 6.25U Dream-Taq Polymerase (Fermentas) and 1 μl of *P. abies* cDNA. Initial denaturation was at 95 °C for 5 min, followed by 35 cycles of: 15 s at 95 °C, 20 s at 58 °C and 120 s at 72 °C and a final elongation step of 3 min at 72 °C. PCR products were directly purified with the GenJet™ PCR-purification kit. Purified PCR products were then cloned into pDONR™/Zeo vectors by Gateway® BP recombination. TOP10 competent cells were transformed and colonies were selected in LB medium with 50 μg/mL zeocin. Colonies were grown overnight on liquid LB medium with 50 μg/mL zeocin and plasmids were isolated using GenJet™ plasmid minikit and plasmids were verified by PCR using the Attb primers for the different G-protein subunits.

*PaGPA1, PaHGB1, PaGG1, PaGG2, PaGG3* and *PaGG4* were transferred from pDONR/Zeo entry vectors into pDest-DB and pDest-AD-CYH2 vectors by Gateway® LR recombination to generate Gal4 DNA binding domain (DB) and Gal4 activation domain (AD) hybrid proteins, respectively. The LR reaction was used to transform into TOP10 competent cells and colonies were selected on LB plates with 100 μg/mL ampicillin. Colonies were grown overnight on liquid LB medium with 100 μg/mL ampicillin and plasmids were isolated using GenJet™ plasmid minikit and plasmids were sequenced at Macrogen (Amsterdam, Netherlands) for confirmation.

The resulted DB and AD plasmids were individually transformed into haploid yeast (*S. cerevisiae*) strains Y8930 (MATα) and Y8800 (MATa) to create baits and preys, respectively as described [63]. Briefly, Y8930 and Y8800 strains were grown in liquid YEPD overnight. A 0.1 OD culture was prepared the following morning. Once the OD reached 0.4-0.6, the cells were harvested and prepared for transformation. The baits and preys were selected on Difco™ yeast nitrogen base (YNB) with leucine dropout (−L) and tryptophan dropout (−T) selective media respectively. The haploid bait and prey yeast strains were pairwise mated o/n in YEPD. The diploid yeast cells were selected onto YNB -LT selective liquid media, and subsequently spotted onto YNB -LTH as well as -LH containing cycloheximide (CHX) selective media. In addition we also determined the strength of protein-protein interaction by supplementing –LTH and -LH with 3-Amino- 1, 2, 4-trizole (3AT), a competitive inhibitor of histidine biosynthesis. Yeast growth on –LTH but not on -LH containing CHX media were scored as positive interactions. Yeast growth found on both –LTH and –LH containing CHX were due to *de* novo autoactivation and hence removed from the data set.

## Availability of supporting data

The data sets supporting the results of this article are included within the article and its additional files.

## Additional files

Additional file 1:
**Accession numbers for the Gα-, Gβ- and** **Gγ-subunits** **sequences found in the plant kingdom.** Sequences have been either cloned or retrieved from the Gene Index Project (The Gene Index Databases-Dana Faber Cancer Institute; Lee et al., [54]; Pertea et al., [55]; Quackenbush et al., [56] ; Tsai et al., [64]), NCBI EST/Nucleotide/Protein, Phytozome v9.1 (Goodstein et al., [57]) or Uniprot [65]; underlined sequences were used for primer design. (DOCX 18 kb) Additional file 2:
**Predictions of** **alpha-helices** **in short** **Gγ-subunits** **PaGG1, PaGG2, GG1 and GG2.** The GGL domain (pfam00631) is underlined. The PreSSAPro software (http://bioinformatica.isa.cnr.it/PRESSAPRO/) was used to predict the formation of α-helices based on amino acid propensities, residues likely to form α-helices are shown in bold font and are boxed, residues shared between three or more proteins are shaded and asterisks indicate conserved amino acids. (PDF 10 kb) Additional file 3:
**Alignment of the** **Gα-subunit** **protein sequences used for the** **Gα-phylogeny;** **the asteriks indicates sequences obtained from Urano et al.** ([9, 10]). (XLS 103 kb) Additional file 4:
**Alignment the** **Gβ-subunit** **protein sequences used for the** **Gβ-phylogeny;** **the asteriks indicates sequences where the accession number corresponds to a nucleotide sequence, instead of an amino acid sequence.** Nucleotide sequences have been translated using the translate function from the Sequence Manipulation Suite [58]. (XLS 105 kb) Additional file 5:
**Phylogeny of Gα- and** **Gβ-subunits** **of the plant kingdom.** The figure shows neighbor-joining trees of full-length sequence alignments of the Gα- (a) and Gβ-subunits. (b) including sequences from species of the Brassicaceae (blue), Fabaceae (orange) and Pinaceae (purple) and the moss *Physcomitrella patens* (green, root). Bootstrap support over 65 is indicated at the nodes. (PDF 502 kb) Additional file 6:
**Alignment of** **Gy-subunit** **sequences.** (PDF 8038 kb) Additional file 7:
**Relative expression values of** ***PaGG1, PaGG3, PaHGB1*** **and** ***PaGPA1*** **in cotyledons and roots of** ***P. abies*** **treated with abscisic acid, methyl jasmonate, wounding at 4 and 24 h** ***post*** **treatment compared to 0 h.** (XLSX 12 kb) Additional file 8:
**Primer sequences for** **full-length** **amplification of the Gα-, Gβ- and** **Gγ-subunits** **cloning of the sequences and qPCR.** (DOCX 17 kb)
