# Supplementary material for: Heterotrimeric G–proteins in Picea abies and their regulation in response to Heterobasidion annosum s.l. infection
Source: BMC Plant Biol. 2015 Dec 12;15:287. doi: 10.1186/s12870-015-0676-1 (PMC4676809; doi:10.1186/s12870-015-0676-1)
Supplement: Additional file 1: — Accession numbers for the Gα-, Gβ- and Gγ-subunits sequences found in the plant kingdom. Sequences have been either cloned or retrieved from the Gene Index Project (The Gene Index Databases-Dana Faber Cancer Institute; Lee et al., [54]; Pertea et al., [55]; Quackenbush et al., [56] ; Tsai et al., [64]), NCBI EST/Nucleotide/Protein, Phytozome v9.1 (Goodstein et al., [57]) or Uniprot [65]; underlined sequences were used for primer design. (DOCX 18 kb) [file 12870_2015_676_MOESM1_ESM.docx]

Additional file 1 Accession numbers for the G**α-**, Gβ- and G**γ**-subunit sequences found in the plant kingdom

| **Species** | **Gα-subunit** | **Gβ-subunit** | **Gγ-subunit** |
| --- | --- | --- | --- |
| *Picea abies* | PaGPA1: KM197161 | PaHGB1: KC825350 | PaGG1: KC825351  PaGG2: KC825352  PaGG3: KC825353  PaGG4: KC825354 |
| *Picea sitchensis* | GPA1: ES872547.1 /BT123461.1 | HGB1: EF677922.1 | GG1: ADE76615.1/BT123286.1  GG2: GT123649.1  GG3: ABK26455.1/EF087200.1  GG4:ADE77369.1/BT124093.1 |
| *Picea glauca* | GPA1: Urano *et al.*  (2012a)/BT106624.1 | HGB1: BT107889.1 | GG1: EX389861.1  GG2: BT117176.1  GG3: BT111616.1 |
| *Pinus taeda* | GPA1: Urano *et al.* (2012a) | HGB1: CO175308.1 | GG1: CO158867.1  GG2: DR093846  GG3: DT638145.1 |
| *Pinus contorta* |  |  | GG1: GT252854.1 |
| *Pinus pinaster* |  |  | GG1: BX254993.1/BX680269.1 |
| *Pinus banksiana* | GPA1: GW772588.1/ GW772929.1 | HGB1: GW771196.1 |  |
| *Physcomitrella patens* |  | CAD21857.2 | Pp1s22 182V6.1 (P. patens 1)  Pp1s39 119V6.2 (P. patens 2) |
| *Medicago truncatula* | XP_003588959.1 | XP_003603911.1 | G7ICP9 (A-type)  Medtr2g042200.1 (C-type 1)  Medtr4g125190.1 (C-type 2)  Medtr8g021170.1 (C-type 3) |
| *Phaseolus vulgaris* | Phvul.001G092700  Phvul.009G082900 |  | Phvul.001G057600.1  Phvul.003G130200.1  Phvul.007G175700.1  Phvul.007G111000.1 |
| *Lotus japonicus* | P49082 | AFK33707.1 | I3SQN8 (B-type)  I3SXY5 (A-type 2)  I3T376 (A-type 1) |
| *Vigna unguiculata* |  | FG808128.1 | FF393368.1  FF545146.1 |
| *Brassica rapa* |  |  | Bra007741  Bra023782 |
| *Brassica napus* | ACX43273.1 | ACX43274.1 | EE552948.1  ACX43275.1 |
| *Capsella rubella* |  |  | Carubv10002620m  Carubv10014985m  Carubv10018305m |
| *Thellungiella halophila* | AET34449.1 | BAJ34135.1 | Thhalv10014548m  Thhalv10006334m  Thhalv10021823m |
| *Arabidopsis lyrata* subsp*. lyrata* | XP_002878953.1 | XP_002867119.1 | XP 002874008.1  XP 002876721.1  XP 002883385.1 |
| *Arabidopsis thaliana* | GPA1: ACX43273.1 | AGB1: AEE86381.1 | AGG1: NP 567147.1  AGG2: NP 850746.1  AGG3: AT5G20635.1 |
| *Raphanus sativus* |  | EY938503.1 | FD578839.1 |
| *Pisum sativum* | AAB57825.1 | AAD49742.1 |  |
| *Glycine max* |  |  | Glyma07g04510.1  Glyma02g16190.1  Glyma14g17060.1  Glyma10g03610.1  Glyma15g19630.1  Glyma17g05640.1  Glyma17g29590.1 |
| *Populus trichocarpa* |  |  | B9H7G6  B9H8E8  POPTR 0006s14330.1  POPTR 0018s07510.1  POPTR 0015s13280.1  POPTR 0002s08200.1  POPTR 0002s04770.1 |
| *Cucumis sativis* |  |  | Cucsa.016430.1  Cucsa.310840.1  Cucsa.386730.1  Cucsa.016430.1  Cucsa.117350.1  Cucsa.141810.1  Cucsa.141810.2  Cucsa.254740.1  Cucsa.310840.1  Cucsa.386730.1 |
| *Ricinus communis* |  |  | 29648.m002019  29844.m003197 |
| *Vitis vinifera* |  |  | GSVIVT01015067001  GSVIVT01018076001 |
| *Manihot esculenta* |  |  | cassava4.1 024861m  cassava4.1 028296m  cassava4.1 019921m  cassava4.1 019686m  cassava4.1 019797m  cassava4.1 023456m  cassava4.1 026181m  cassava4.1 014412m |
| *Zea mays* |  |  | NP 001151842.1  B6U329  B6TUI6 |
| *Oryza sativa subsp. indica* |  |  | RGG1: B8AN27  RGG2: ACY05516.1 |
| *Sorghum bicolor* |  |  | C5XUI0  C5XDQ6  XP 002465152.1  XP 002444469.1  XP 002460275.1 |
| *Brachypodium distachyon* |  |  | I1GQ51  I1HX13 |

Sequences have been either cloned or retrieved from the Gene Index Project (Dana Faber Cancer Institute; Lee et al., 2002; Pertea et al., 2003; Quackenbush et al., 2001; Tsai et al., 2001), NCBI EST/Nucleotide/Protein, Phytozome v9.1 (Goodstein et al., 2012), Uniprot (The Uniprot Consortium, 2012) and/or Urano et al. 2012a
