# Supplementary material for: Heterotrimeric G–proteins in Picea abies and their regulation in response to Heterobasidion annosum s.l. infection
Source: BMC Plant Biol. 2015 Dec 12;15:287. doi: 10.1186/s12870-015-0676-1 (PMC4676809; doi:10.1186/s12870-015-0676-1)
Supplement: Additional file 2: — Predictions of alpha-helices in short Gγ-subunits PaGG1, PaGG2, GG1 and GG2. The GGL domain (pfam00631) is underlined. The PreSSAPro software (http://bioinformatica.isa.cnr.it/PRESSAPRO/) was used to predict the formation of α-helices based on amino acid propensities, residues likely to form α-helices are shown in bold font and are boxed, residues shared between three or more proteins are shaded and asterisks indicate conserved amino acids. (PDF 10 kb) [file 12870_2015_676_MOESM2_ESM.pdf]

```
        *
AtGG2   M E A G - - - - - - - S S N S S G Q L S G R V V D T R - - - - -   [ 32]
AtGG1   M R E E T V V Y E Q E E S V S H G - - - - - - - - - - - - - - -   [ 32]
PaGG1   M E E E T - - - - - D T S V S T G A Q N G R P Q D S K Q P P T E   [ 32]
PaGG2   M Q G T T R W I S - - - - - - - - - - - D M K R L E P R P T P V A [ 32]

                  *                 *          *          * *    * *
AtGG2   - - - - G K H R I Q A E L K R L E Q E A R F L E E E L E Q L E K   [ 64]
AtGG1   - - - G G K H R I L A E L A R V E Q E V A F L E K E L K E V E N   [ 64]
PaGG1   T D V G G K H R K L A E L H R L N Q E I R F L E E E L E D L D K   [ 64]
PaGG2   P N L R G I Q H R L S Q L N H L E Q Q I K L L E D E L E E L H N   [ 64]

                    *       *               * *     *     *   * *   *
AtGG2   M D N A S A S C K E F L D S V D S K P D P L L P E T T G P V N A   [ 96]
AtGG1   T D I V S T V C E E L L S V I E K G P D P L L P L T N G P L N L   [ 96]
PaGG1   I D K A T S A C K E M L L I I E N T P D P L L S V T K G P E N P   [ 96]
PaGG2   T D R A S T V C R D V L M T V D S R P D A F L P S T I G P E N A   [ 96]

          *     *   *               *   *
AtGG2   T W D Q W F E G P K E A K R C G C S I L   [116]
AtGG1   G W D R W F E G P N G G E G C R C L I L   [116]
PaGG1   A W D R W F E G P V E S D G C K C W I I   [116]
PaGG2   A W K Q W L E K T T E S R G C G C C L -   [116]
```
