# Supplementary figures and images for: Heterotrimeric G–proteins in Picea abies and their regulation in response to Heterobasidion annosum s.l. infection
Source: BMC Plant Biol. 2015 Dec 12;15:287. doi: 10.1186/s12870-015-0676-1 (PMC4676809; doi:10.1186/s12870-015-0676-1)

a

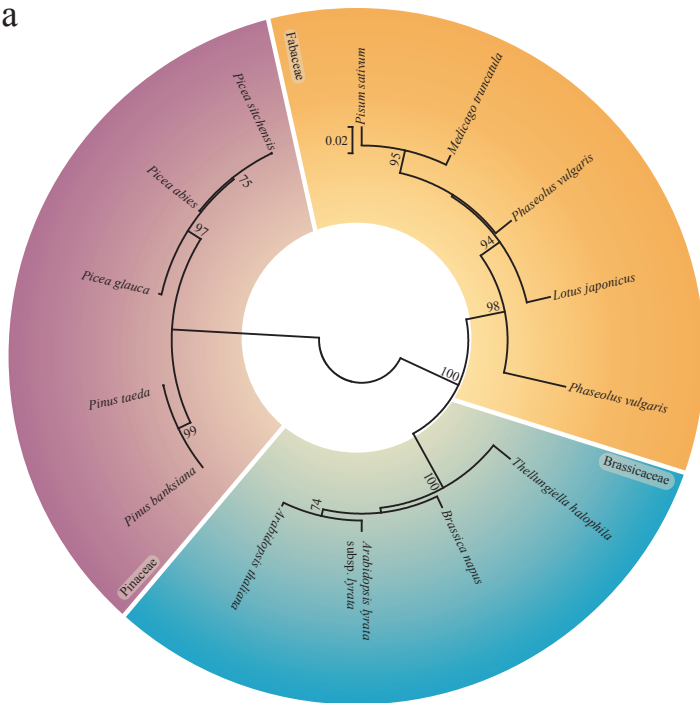

b

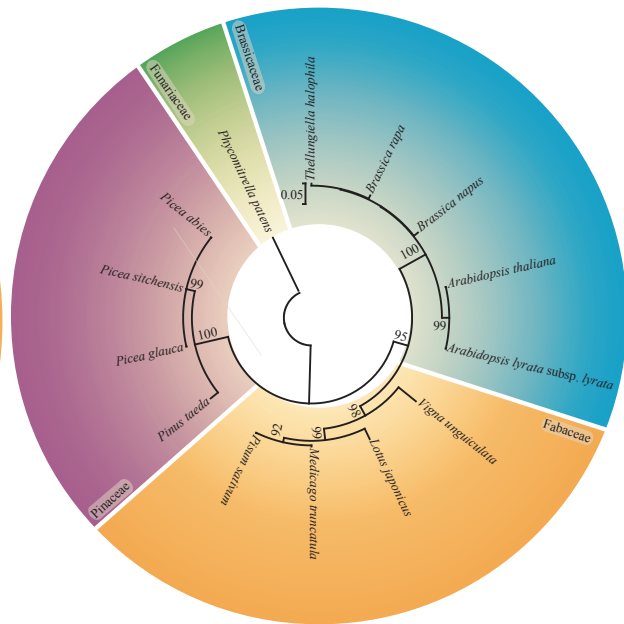

Supplement: Additional file 5: — Phylogeny of Gα- and Gβ-subunits of the plant kingdom. The figure shows neighbor-joining trees of full-length sequence alignments of the Gα- (a) and Gβ-subunits. (b) including sequences from species of the Brassicaceae (blue), Fabaceae (orange) and Pinaceae (purple) and the moss Physcomitrella patens (green, root). Bootstrap support over 65 is indicated at the nodes. (PDF 502 kb) [file 12870_2015_676_MOESM5_ESM.pdf]
