# Supplementary material for: Heterotrimeric G–proteins in Picea abies and their regulation in response to Heterobasidion annosum s.l. infection
Source: BMC Plant Biol. 2015 Dec 12;15:287. doi: 10.1186/s12870-015-0676-1 (PMC4676809; doi:10.1186/s12870-015-0676-1)
Supplement: Additional file 6: — Alignment of Gy-subunit sequences. (PDF 8038 kb) [file 12870_2015_676_MOESM6_ESM.pdf]

a

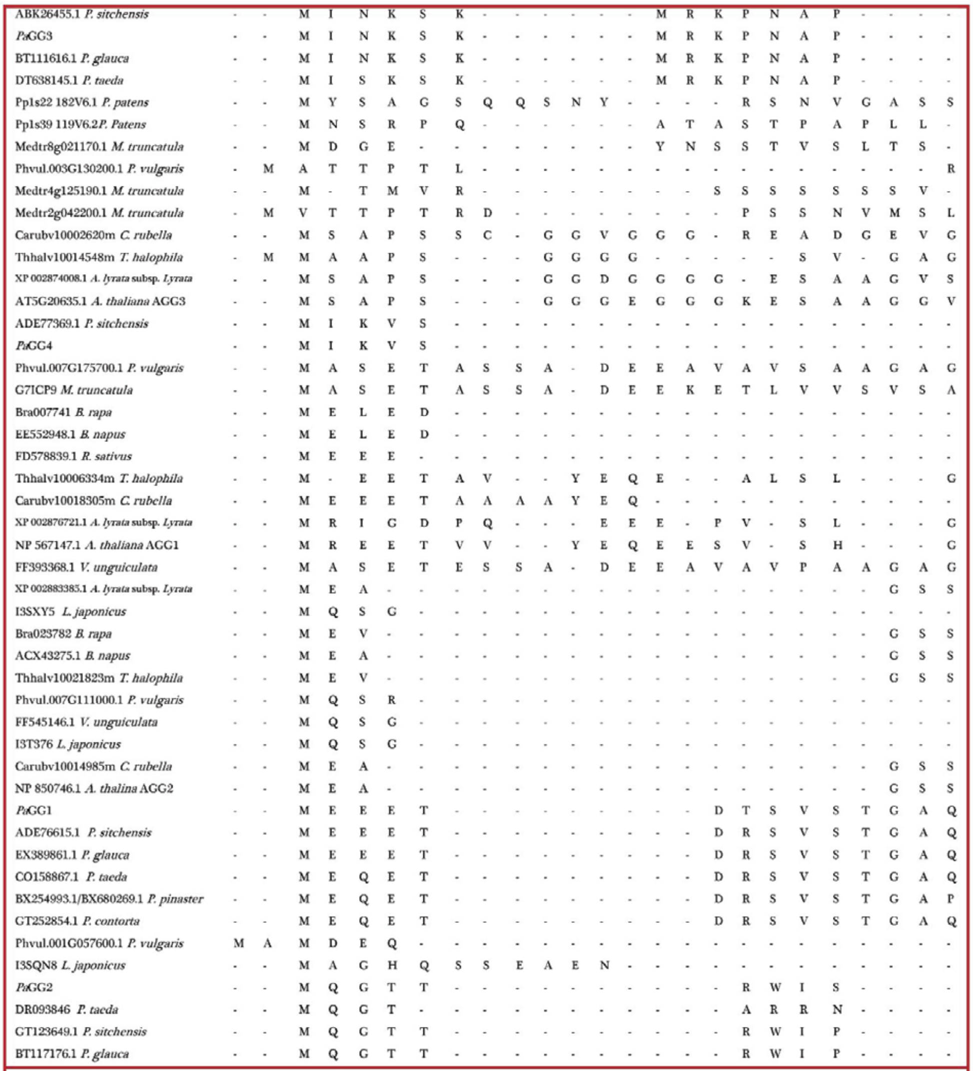
ABK26455.1 *P. sitchensis* - - M I N K S K - - - - - - M R K P N A P - - - -
*Pa*GG3 - - M I N K S K - - - - - - M R K P N A P - - - -
BT111616.1 *P. glauca* - - M I N K S K - - - - - - M R K P N A P - - - -
DT638145.1 *P. taeda* - - M I S K S K - - - - - - M R K P N A P - - - -
Pp1s22 182V6.1 *P. patens* - - M Y S A G S Q Q S N Y - - - - R S N V G A S S
Pp1s39 119V6.2*P. Patens* - - M N S R P Q - - - - - - A T A S T P A P L L -
Medtr8g021170.1 *M. truncatula* - - M D G E - - - - - - - - Y N S S T V S L T S -
Phvul.003G130200.1 *P. vulgaris* - M A T T P T L - - - - - - - - - - - - - - - - R
Medtr4g125190.1 *M. truncatula* - - M - T M V R - - - - - - - - S S S S S S S V -
Medtr2g042200.1 *M. truncatula* - M V T T P T R D - - - - - - - - P S S N V M S L
Carubv10002620m *C. rubella* - - M S A P S S C - G G V G G G - R E A D G E V G
Thhalv10014548m *T. halophila* - M M A A P S - - - G G G G - - - - - S V - G A G
XP 002874008.1 *A. lyrata* subsp. *Lyrata* - - M S A P S - - - G G D G G G G - E S A A G V S
AT5G20635.1 *A. thaliana* AGG3 - - M S A P S - - - G G G E G G G K E S A A G G V
ADE77369.1 *P. sitchensis* - - M I K V S - - - - - - - - - - - - - - - - - -
*Pa*GG4 - - M I K V S - - - - - - - - - - - - - - - - - -
Phvul.007G175700.1 *P. vulgaris* - - M A S E T A S S A - D E E A V A V S A A G A G
G7ICP9 *M. truncatula* - - M A S E T A S S A - D E E K E T L V V S V S A
Bra007741 *B. rapa* - - M E L E D - - - - - - - - - - - - - - - - - -
EE552948.1 *B. napus* - - M E L E D - - - - - - - - - - - - - - - - - -
FD578839.1 *R. sativus* - - M E E E - - - - - - - - - - - - - - - - - - -
Thhalv10006334m *T. halophila* - - M - E E T A V - - Y E Q E - - A L S L - - - G
Carubv10018305m *C. rubella* - - M E E E T A A A A Y E Q - - - - - - - - - - -
XP 002876721.1 *A. lyrata* subsp. *Lyrata* - - M R I G D P Q - - - E E E - P V - S L - - - G
NP 567147.1 *A. thaliana* AGG1 - - M R E E T V V - - Y E Q E E S V - S H - - - G
FF393368.1 *V. unguiculata* - - M A S E T E S S A - D E E A V A V P A A G A G
XP 002883385.1 *A. lyrata* subsp. *Lyrata* - - M E A - - - - - - - - - - - - - - - - - G S S
I3SXY5 *L. japonicus* - - M Q S G - - - - - - - - - - - - - - - - - - -
Bra023782 *B. rapa* - - M E V - - - - - - - - - - - - - - - - - G S S
ACX43275.1 *B. napus* - - M E A - - - - - - - - - - - - - - - - - G S S
Thhalv10021823m *T. halophila* - - M E V - - - - - - - - - - - - - - - - - G S S
Phvul.007G111000.1 *P. vulgaris* - - M Q S R - - - - - - - - - - - - - - - - - - -
FF545146.1 *V. unguiculata* - - M Q S G - - - - - - - - - - - - - - - - - - -
I3T376 *L. japonicus* - - M Q S G - - - - - - - - - - - - - - - - - - -
Carubv10014985m *C. rubella* - - M E A - - - - - - - - - - - - - - - - - G S S
NP 850746.1 *A. thalina* AGG2 - - M E A - - - - - - - - - - - - - - - - - G S S
*Pa*GG1 - - M E E E T - - - - - - - - - D T S V S T G A Q
ADE76615.1 *P. sitchensis* - - M E E E T - - - - - - - - - D R S V S T G A Q
EX389861.1 *P. glauca* - - M E E E T - - - - - - - - - D R S V S T G A Q
CO158867.1 *P. taeda* - - M E Q E T - - - - - - - - - D R S V S T G A Q
BX254993.1/BX680269.1 *P. pinaster* - - M E Q E T - - - - - - - - - D R S V S T G A P
GT252854.1 *P. contorta* - - M E Q E T - - - - - - - - - D R S V S T G A Q
Phvul.001G057600.1 *P. vulgaris* M A M D E Q - - - - - - - - - - - - - - - - - - -
I3SQN8 *L. japonicus* - - M A G H Q S S E A E N - - - - - - - - - - - -
*Pa*GG2 - - M Q G T T - - - - - - - - - - R W I S - - - -
DR093846 *P. taeda* - - M Q G T - - - - - - - - - - - A R R N - - - -
GT123649.1 *P. sitchensis* - - M Q G T T - - - - - - - - - - R W I P - - - -
BT117176.1 *P. glauca* - - M Q G T T - - - - - - - - - - R W I P - - - -

b

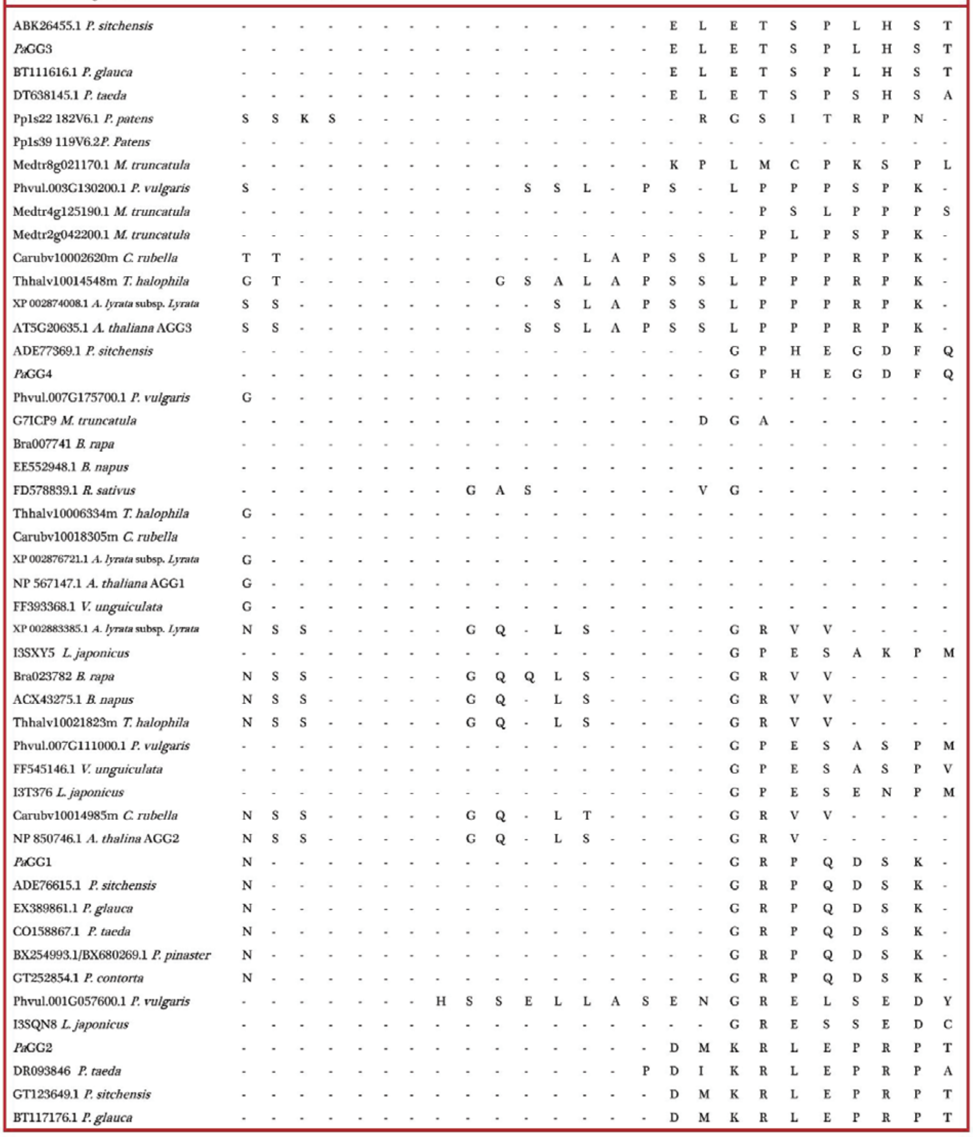
ABK26455.1 *P. sitchensis* - - - - - - - - - - - - - - - E L E T S P L H S T
*Pa*GG3 - - - - - - - - - - - - - - - E L E T S P L H S T
BT111616.1 *P. glauca* - - - - - - - - - - - - - - - E L E T S P L H S T
DT638145.1 *P. taeda* - - - - - - - - - - - - - - - E L E T S P S H S A
Pp1s22 182V6.1 *P. patens* S S K S - - - - - - - - - - - - R G S I T R P N -
Pp1s39 119V6.2*P. Patens* -
Medtr8g021170.1 *M. truncatula* - - - - - - - - - - - - - - - K P L M C P K S P L
Phvul.003G130200.1 *P. vulgaris* S - - - - - - - - - S S L - P S - L P P P S P K -
Medtr4g125190.1 *M. truncatula* - - - - - - - - - - - - - - - - - - P S L P P P S
Medtr2g042200.1 *M. truncatula* - - - - - - - - - - - - - - - - - - P L P S P K -
Carubv10002620m *C. rubella* T T - - - - - - - - - - L A P S S L P P P R P K -
Thhalv10014548m *T. halophila* G T - - - - - - - G S A L A P S S L P P P R P K -
XP 002874008.1 *A. lyrata* subsp. *Lyrata* S S - - - - - - - - - S L A P S S L P P P R P K -
AT5G20635.1 *A. thaliana* AGG3 S S - - - - - - - - S S L A P S S L P P P R P K -
ADE77369.1 *P. sitchensis* - - - - - - - - - - - - - - - - - G P H E G D F Q
*Pa*GG4 - - - - - - - - - - - - - - - - - G P H E G D F Q
Phvul.007G175700.1 *P. vulgaris* G -
G7ICP9 *M. truncatula* - - - - - - - - - - - - - - - - D G A - - - - - -
Bra007741 *B. rapa* -
EE552948.1 *B. napus* -
FD578839.1 *R. sativus* - - - - - - - - G A S - - - - - V G - - - - - - -
Thhalv10006334m *T. halophila* G -
Carubv10018305m *C. rubella* -
XP 002876721.1 *A. lyrata* subsp. *Lyrata* G -
NP 567147.1 *A. thaliana* AGG1 G -
FF393368.1 *V. unguiculata* G -
XP 002883385.1 *A. lyrata* subsp. *Lyrata* N S S - - - - - G Q - L S - - - - G R V V - - - -
I3SXY5 *L. japonicus* - - - - - - - - - - - - - - - - - G P E S A K P M
Bra023782 *B. rapa* N S S - - - - - G Q Q L S - - - - G R V V - - - -
ACX43275.1 *B. napus* N S S - - - - - G Q - L S - - - - G R V V - - - -
Thhalv10021823m *T. halophila* N S S - - - - - G Q - L S - - - - G R V V - - - -
Phvul.007G111000.1 *P. vulgaris* - - - - - - - - - - - - - - - - - G P E S A S P M
FF545146.1 *V. unguiculata* - - - - - - - - - - - - - - - - - G P E S A S P V
I3T376 *L. japonicus* - - - - - - - - - - - - - - - - - G P E S E N P M
Carubv10014985m *C. rubella* N S S - - - - - G Q - L T - - - - G R V V - - - -
NP 850746.1 *A. thalina* AGG2 N S S - - - - - G Q - L S - - - - G R V - - - - -
*Pa*GG1 N - - - - - - - - - - - - - - - - G R P Q D S K -
ADE76615.1 *P. sitchensis* N - - - - - - - - - - - - - - - - G R P Q D S K -
EX389861.1 *P. glauca* N - - - - - - - - - - - - - - - - G R P Q D S K -
CO158867.1 *P. taeda* N - - - - - - - - - - - - - - - - G R P Q D S K -
BX254993.1/BX680269.1 *P. pinaster* N - - - - - - - - - - - - - - - - G R P Q D S K -
GT252854.1 *P. contorta* N - - - - - - - - - - - - - - - - G R P Q D S K -
Phvul.001G057600.1 *P. vulgaris* - - - - - - - H S S E L L A S E N G R E L S E D Y
I3SQN8 *L. japonicus* - - - - - - - - - - - - - - - - - G R E S S E D C
*Pa*GG2 - - - - - - - - - - - - - - - D M K R L E P R P T
DR093846 *P. taeda* - - - - - - - - - - - - - - P D I K R L E P R P A
GT123649.1 *P. sitchensis* - - - - - - - - - - - - - - - D M K R L E P R P T
BT117176.1 *P. glauca* - - - - - - - - - - - - - - - D M K R L E P R P T

c

ABK26455.1 *P. sitchensis*	R	H	-	-	-	-	-	C	A	Y	P	-	-	-	-	-	-	-	-	-	-	-	-	-	-
*Pa*GG3	R	H	-	-	-	-	-	C	A	Y	P	-	-	-	-	-	-	-	-	-	-	-	-	-	-
BT111616.1 *P. glauca*	R	H	-	-	-	-	-	C	A	Y	P	-	-	-	-	-	-	-	-	-	-	-	-	-	-
DT638145.1 *P. taeda*	R	H	-	-	-	-	-	C	A	Y	P	-	-	-	-	-	-	-	-	-	-	-	-	-	-
Pp1s22 182V6.1 *P. patens*	P	T	P	P	-	-	-	-	-	-	-	-	-	-	-	-	-	-	-	-	-	-	-	-	-
Pp1s39 119V6.2*P. Patens*	-	-	-	-	-	-	-	-	-	-	-	-	-	-	-	-	-	-	-	-	-	-	-	-	-
Medtr8g021170.1 *M. truncatula*	P	G	F	-	-	-	-	-	-	-	-	-	-	-	-	-	-	-	-	-	-	-	-	-	-
Phvul.003G130200.1 *P. vulgaris*	-	-	-	-	S	P	P	-	E	Y	P	-	-	-	-	-	-	-	-	-	-	-	-	-	-
Medtr4g125190.1 *M. truncatula*	P	K	-	-	S	P	P	-	E	Y	P	-	-	-	-	-	-	-	-	-	-	-	-	-	-
Medtr2g042200.1 *M. truncatula*	-	-	-	-	A	P	P	-	E	Y	P	-	-	-	-	-	-	-	-	-	-	-	-	-	-
Carubv10002620m *C. rubella*	-	-	-	-	S	P	P	-	E	Y	P	-	-	-	-	-	-	-	-	-	-	-	-	-	-
Thhalv10014548m *T. halophila*	-	-	-	-	S	P	P	-	E	Y	P	-	-	-	-	-	-	-	-	-	-	-	-	-	-
XP 002874008.1 *A. lyrata* subsp. *Lyrata*	-	-	-	-	S	P	P	-	E	Y	P	-	-	-	-	-	-	-	-	-	-	-	-	-	-
AT5G20635.1 *A. thaliana* AGG3	-	-	-	-	S	P	P	-	E	Y	P	-	-	-	-	-	-	-	-	-	-	-	-	-	-
ADE77369.1 *P. sitchensis*	W	V	V	V	Y	R	-	-	-	-	-	-	-	-	-	-	-	-	-	-	-	-	-	-	-
*Pa*GG4	W	V	V	V	Y	R	-	-	-	-	-	-	-	-	-	-	-	-	-	-	-	-	-	-	-
Phvul.007G175700.1 *P. vulgaris*	-	-	-	-	-	-	-	-	-	-	-	-	-	-	-	-	-	-	-	-	-	-	-	-	-
G7ICP9 *M. truncatula*	-	-	-	-	-	-	-	-	-	-	-	-	-	-	-	-	-	-	-	-	-	-	-	-	-
Bra007741 *B. rapa*	-	-	-	-	-	-	-	-	-	-	-	-	-	-	-	-	-	-	-	-	-	-	-	-	-
EE552948.1 *B. napus*	-	-	-	-	-	-	-	-	-	-	-	-	-	-	-	-	-	-	-	-	-	-	-	-	-
FD578839.1 *R. sativus*	-	-	-	-	-	-	-	-	-	-	-	-	-	-	-	-	-	-	-	-	-	-	-	-	-
Thhalv10006334m *T. halophila*	-	-	-	-	-	-	-	-	-	-	-	-	-	-	-	-	-	-	-	-	-	-	-	-	-
Carubv10018305m *C. rubella*	-	-	-	-	-	-	-	-	-	-	-	-	-	-	-	-	-	-	-	-	-	-	-	-	-
XP 002876721.1 *A. lyrata* subsp. *Lyrata*	-	-	-	-	-	-	-	-	-	-	-	-	-	-	-	-	-	-	-	-	-	-	-	-	-
NP 567147.1 *A. thaliana* AGG1	-	-	-	-	-	-	-	-	-	-	-	-	-	-	-	-	-	-	-	-	-	-	-	-	-
FF393368.1 *V. unguiculata*	-	-	-	-	-	-	-	-	-	-	-	-	-	-	-	-	-	-	-	-	-	-	-	-	-
XP 002883385.1 *A. lyrata* subsp. *Lyrata*	-	-	-	-	-	-	-	-	-	-	-	-	-	-	-	-	-	-	-	-	-	-	-	-	-
I3SXY5 *L. japonicus*	-	-	-	-	-	-	-	-	-	T	H	R	T	H	S	Q	S	S	-	-	-	-	-	-	-
Bra023782 *B. rapa*	-	-	-	-	-	-	-	-	-	-	-	-	-	-	-	-	-	-	-	-	-	-	-	-	-
ACX43275.1 *B. napus*	-	-	-	-	-	-	-	-	-	-	-	-	-	-	-	-	-	-	-	-	-	-	-	-	-
Thhalv10021823m *T. halophila*	-	-	-	-	-	-	-	-	-	-	-	-	-	-	-	-	-	-	-	-	-	-	-	-	-
Phvul.007G111000.1 *P. vulgaris*	-	-	-	-	-	-	-	-	-	T	H	R	V	Q	S	L	S	S	-	-	-	-	-	-	-
FF545146.1 *V. unguiculata*	-	-	-	-	-	-	-	-	-	T	H	R	V	Q	S	L	S	S	-	-	-	-	-	-	-
I3T376 *L. japonicus*	T	-	-	-	-	-	-	-	-	-	E	R	A	Q	S	L	S	S	-	-	-	-	-	-	-
Carubv10014985m *C. rubella*	-	-	-	-	-	-	-	-	-	-	-	-	-	-	-	-	-	-	-	-	-	-	-	-	-
NP 850746.1 *A. thalina* AGG2	-	-	-	-	-	-	-	-	-	-	-	-	-	-	-	-	-	-	-	-	-	-	-	-	-
*Pa*GG1	-	-	-	-	-	-	Q	P	P	T	E	-	-	-	-	-	-	-	-	-	-	-	-	-	-
ADE76615.1 *P. sitchensis*	-	-	-	-	-	-	Q	P	P	T	E	-	-	-	-	-	-	-	-	-	-	-	-	-	-
EX389861.1 *P. glauca*	-	-	-	-	-	-	Q	P	P	T	E	-	-	-	-	-	-	-	-	-	-	-	-	-	-
CO158867.1 *P. taeda*	-	-	-	-	-	-	Q	P	P	T	E	-	-	-	-	-	-	-	-	-	-	-	-	-	-
BX254993.1/BX680269.1 *P. pinaster*	-	-	-	-	-	-	Q	P	P	T	E	-	-	-	-	-	-	-	-	-	-	-	-	-	-
GT252854.1 *P. contorta*	-	-	-	-	-	-	Q	P	P	T	E	-	-	-	-	-	-	-	-	-	-	-	-	-	-
Phvul.001G057600.1 *P. vulgaris*	R	E	E	G	T	-	Q	K	G	S	H	H	P	Q	S	G	T	T	S	L	P	A	-	-	-
I3SQN8 *L. japonicus*	R	E	R	D	P	A	-	-	-	-	-	H	P	L	S	Q	T	-	-	-	-	-	-	-	-
*Pa*GG2	P	V	A	P	-	-	-	-	-	-	-	-	-	-	-	-	-	-	-	-	-	-	-	-	-
DR093846 *P. taeda*	P	G	T	P	-	-	-	-	-	-	-	-	-	-	-	-	-	-	-	-	-	-	-	-	-
GT123649.1 *P. sitchensis*	P	G	A	P	-	-	-	-	-	-	-	-	-	-	-	-	-	-	-	-	-	-	-	-	-
BT117176.1 *P. glauca*	P	G	A	P	-	-	-	-	-	-	-	-	-	-	-	-	-	-	-	-	-	-	-	-	-

d

ABK26455.1 *P. sitchensis*	-	-	-	-	-	-	-	-	-	-	-	-	-	-	-	-	-	-	-	-	-	-	-	-	-
*Pa*GG3	-	-	-	-	-	-	-	-	-	-	-	-	-	-	-	-	-	-	-	-	-	-	-	-	-
BT111616.1 *P. glauca*	-	-	-	-	-	-	-	-	-	-	-	-	-	-	-	-	-	-	-	-	-	-	-	-	-
DT638145.1 *P. taeda*	-	-	-	-	-	-	-	-	-	-	-	-	-	-	-	-	-	-	-	-	-	-	-	-	-
Pp1s22 182V6.1 *P. patens*	-	-	-	-	-	-	-	-	-	-	-	-	-	-	-	-	M	T	P	L	R	Q	E	-	A
Pp1s39 119V6.2*P. Patens*	-	-	-	-	-	-	-	-	-	-	-	-	-	-	-	-	-	-	-	-	-	-	-	-	-
Medtr8g021170.1 *M. truncatula*	-	-	-	-	-	-	-	-	-	-	-	-	-	-	-	-	-	-	-	-	-	-	-	-	-
Phvul.003G130200.1 *P. vulgaris*	-	-	-	-	-	-	-	-	-	-	-	-	-	-	-	-	-	-	-	-	-	-	-	-	-
Medtr4g125190.1 *M. truncatula*	-	-	-	-	-	-	-	-	-	-	-	-	-	-	-	-	-	-	-	-	-	-	-	-	-
Medtr2g042200.1 *M. truncatula*	-	-	-	-	-	-	-	-	-	-	-	-	-	-	-	-	-	-	-	-	-	-	-	-	-
Carubv10002620m *C. rubella*	-	-	-	-	-	-	-	-	-	-	-	-	-	-	-	-	-	-	-	-	-	-	-	-	-
Thhalv10014548m *T. halophila*	-	-	-	-	-	-	-	-	-	-	-	-	-	-	-	-	-	-	-	-	-	-	-	-	-
XP 002874008.1 *A. lyrata* subsp. *Lyrata*	-	-	-	-	-	-	-	-	-	-	-	-	-	-	-	-	-	-	-	-	-	-	-	-	-
AT5G20635.1 *A. thaliana* AGG3	-	-	-	-	-	-	-	-	-	-	-	-	-	-	-	-	-	-	-	-	-	-	-	-	-
ADE77369.1 *P. sitchensis*	-	R	G	V	H	I	D	V	L	E	E	R	M	Q	A	V	G	G	H	V	G	V	D	S	T
*Pa*GG4	-	R	G	V	H	I	D	V	L	E	E	R	M	Q	A	V	G	G	H	V	G	V	D	S	T
Phvul.007G175700.1 *P. vulgaris*	-	-	-	-	-	-	-	-	-	-	-	-	-	-	-	-	-	-	-	-	-	-	-	-	-
G7ICP9 *M. truncatula*	-	-	-	-	-	-	-	-	-	-	-	-	-	-	-	-	-	-	-	-	-	-	-	-	-
Bra007741 *B. rapa*	-	-	-	-	-	-	-	-	-	-	-	-	-	-	-	-	-	-	-	-	-	-	-	-	-
EE552948.1 *B. napus*	-	-	-	-	-	-	-	-	-	-	-	-	-	-	-	-	-	-	-	-	-	-	-	-	-
FD578839.1 *R. sativus*	-	-	-	-	-	-	-	-	-	-	-	-	-	-	-	-	-	-	-	-	-	-	-	-	-
Thhalv10006334m *T. halophila*	-	-	-	-	-	-	-	-	-	-	-	-	-	-	-	-	-	-	-	-	-	-	-	-	-
Carubv10018305m *C. rubella*	-	-	-	-	-	-	-	-	-	-	-	-	-	-	-	-	-	-	-	-	-	-	-	-	-
XP 002876721.1 *A. lyrata* subsp. *Lyrata*	-	-	-	-	-	-	-	-	-	-	-	-	-	-	-	-	-	-	-	-	-	-	-	-	-
NP 567147.1 *A. thaliana* AGG1	-	-	-	-	-	-	-	-	-	-	-	-	-	-	-	-	-	-	-	-	-	-	-	-	-
FF393368.1 *V. unguiculata*	-	-	-	-	-	-	-	-	-	-	-	-	-	-	-	-	-	-	-	-	-	-	-	-	-
XP 002883385.1 *A. lyrata* subsp. *Lyrata*	-	-	-	-	-	-	-	-	-	-	-	-	-	-	-	-	-	-	-	-	-	-	-	-	-
I3SXY5 *L. japonicus*	-	-	-	-	-	-	-	-	-	-	-	-	-	-	-	-	-	-	-	-	-	-	-	-	-
Bra023782 *B. rapa*	-	-	-	-	-	-	-	-	-	-	-	-	-	-	-	-	-	-	-	-	-	-	-	-	-
ACX43275.1 *B. napus*	-	-	-	-	-	-	-	-	-	-	-	-	-	-	-	-	-	-	-	-	-	-	-	-	-
Thhalv10021823m *T. halophila*	-	-	-	-	-	-	-	-	-	-	-	-	-	-	-	-	-	-	-	-	-	-	-	-	-
Phvul.007G111000.1 *P. vulgaris*	-	-	-	-	-	-	-	-	-	-	-	-	-	-	-	-	-	-	-	-	-	-	-	-	-
FF545146.1 *V. unguiculata*	-	-	-	-	-	-	-	-	-	-	-	-	-	-	-	-	-	-	-	-	-	-	-	-	-
I3T376 *L. japonicus*	-	-	-	-	-	-	-	-	-	-	-	-	-	-	-	-	-	-	-	-	-	-	-	-	-
Carubv10014985m *C. rubella*	-	-	-	-	-	-	-	-	-	-	-	-	-	-	-	-	-	-	-	-	-	-	-	-	-
NP 850746.1 *A. thalina* AGG2	-	-	-	-	-	-	-	-	-	-	-	-	-	-	-	-	-	-	-	-	-	-	-	-	-
*Pa*GG1	-	-	-	-	-	-	-	-	-	-	-	-	-	-	-	-	-	-	-	-	-	-	-	-	-
ADE76615.1 *P. sitchensis*	-	-	-	-	-	-	-	-	-	-	-	-	-	-	-	-	-	-	-	-	-	-	-	-	-
EX389861.1 *P. glauca*	-	-	-	-	-	-	-	-	-	-	-	-	-	-	-	-	-	-	-	-	-	-	-	-	-
CO158867.1 *P. taeda*	-	-	-	-	-	-	-	-	-	-	-	-	-	-	-	-	-	-	-	-	-	-	-	-	-
BX254993.1/BX680269.1 *P. pinaster*	-	-	-	-	-	-	-	-	-	-	-	-	-	-	-	-	-	-	-	-	-	-	-	-	-
GT252854.1 *P. contorta*	-	-	-	-	-	-	-	-	-	-	-	-	-	-	-	-	-	-	-	-	-	-	-	-	-
Phvul.001G057600.1 *P. vulgaris*	-	-	-	-	-	-	-	-	-	-	-	-	-	-	-	-	-	-	-	-	-	-	-	-	-
I3SQN8 *L. japonicus*	-	-	-	-	-	-	-	-	-	-	-	-	-	-	-	-	-	-	-	-	-	-	-	-	-
*Pa*GG2	-	-	-	-	-	-	-	-	-	-	-	-	-	-	-	-	-	-	-	-	-	-	-	-	-
DR093846 *P. taeda*	-	-	-	-	-	-	-	-	-	-	-	-	-	-	-	-	-	-	-	-	-	-	-	-	-
GT123649.1 *P. sitchensis*	-	-	-	-	-	-	-	-	-	-	-	-	-	-	-	-	-	-	-	-	-	-	-	-	-
BT117176.1 *P. glauca*	-	-	-	-	-	-	-	-	-	-	-	-	-	-	-	-	-	-	-	-	-	-	-	-	-

e

ABK26455.1 *P. sitchensis*	-	-	-	-	-	-	-	-	-	-	-	-	-	-	-	-	D	I	-	-	-	R	G	R	H
*Pa*GG3	-	-	-	-	-	-	-	-	-	-	-	-	-	-	-	-	D	I	-	-	-	R	G	R	H
BT111616.1 *P. glauca*	-	-	-	-	-	-	-	-	-	-	-	-	-	-	-	-	D	I	-	-	-	R	G	R	H
DT638145.1 *P. taeda*	-	-	-	-	-	-	-	-	-	-	-	-	-	-	-	-	D	I	-	-	-	R	G	K	H
Pp1s22 182V6.1 *P. patens*	I	P	S	L	L	F	H	R	P	S	I	S	A	-	P	S	-	I	-	-	-	R	G	R	S
Pp1s39 119V6.2*P. Patens*	-	-	-	-	-	-	-	-	-	-	-	-	-	-	-	-	-	-	-	-	G	R	G	R	N
Medtr8g021170.1 *M. truncatula*	-	-	-	-	-	-	-	-	-	-	-	-	-	-	-	V	D	F	-	-	-	H	G	K	R
Phvul.003G130200.1 *P. vulgaris*	-	-	-	-	-	-	-	-	-	-	-	-	-	-	-	-	D	L	-	-	-	Y	G	K	R
Medtr4g125190.1 *M. truncatula*	-	-	-	-	-	-	-	-	-	-	-	-	-	-	-	-	D	L	-	-	-	Y	G	K	R
Medtr2g042200.1 *M. truncatula*	-	-	-	-	-	-	-	-	-	-	-	-	-	-	-	-	D	L	-	-	-	Y	G	K	R
Carubv10002620m *C. rubella*	-	-	-	-	-	-	-	-	-	-	-	-	-	-	-	-	D	L	-	-	-	Y	G	K	R
Thhalv10014548m *T. halophila*	-	-	-	-	-	-	-	-	-	-	-	-	-	-	-	-	D	L	-	-	-	Y	G	K	R
XP 002874008.1 *A. lyrata* subsp. *Lyrata*	-	-	-	-	-	-	-	-	-	-	-	-	-	-	-	-	D	L	-	-	-	Y	G	K	R
AT5G20635.1 *A. thaliana* AGG3	-	-	-	-	-	-	-	-	-	-	-	-	-	-	-	-	D	L	-	-	-	Y	G	K	R
ADE77369.1 *P. sitchensis*	S	R	R	G	Q	P	E	P	R	T	P	P	L	H	P	-	D	I	-	-	-	N	G	K	Y
*Pa*GG4	S	R	R	G	Q	P	E	P	R	T	P	P	R	H	P	-	D	I	-	-	-	N	G	K	Y
Phvul.007G175700.1 *P. vulgaris*	-	-	-	-	-	-	-	-	-	-	-	-	-	-	-	T	D	K	-	-	-	R	G	R	H
G7ICP9 *M. truncatula*	-	-	-	-	-	-	-	-	-	-	-	-	-	-	-	T	D	K	-	-	-	R	G	K	H
Bra007741 *B. rapa*	-	-	-	-	-	-	-	-	-	-	-	-	-	-	-	G	D	S	-	-	-	R	G	K	H
EE552948.1 *B. napus*	-	-	-	-	-	-	-	-	-	-	-	-	-	-	-	G	D	A	-	-	-	R	G	K	H
FD578839.1 *R. sativus*	-	-	-	-	-	-	-	-	-	-	-	-	-	-	-	-	D	A	-	-	-	R	G	K	H
Thhalv10006334m *T. halophila*	-	-	-	-	-	-	-	-	-	-	-	-	-	-	-	-	D	A	-	-	-	R	G	K	H
Carubv10018305m *C. rubella*	-	-	-	-	-	-	-	-	-	-	-	-	-	-	-	-	E	A	V	S	L	G	G	K	H
XP 002876721.1 *A. lyrata* subsp. *Lyrata*	-	-	-	-	-	-	-	-	-	-	-	-	-	-	-	-	-	-	-	-	-	-	G	K	H
NP 567147.1 *A. thaliana* AGG1	-	-	-	-	-	-	-	-	-	-	-	-	-	-	-	-	-	-	-	-	-	-	G	K	H
FF393368.1 *V. unguiculata*	-	-	-	-	-	-	-	-	-	-	-	-	-	-	-	T	D	K	-	-	-	R	G	R	H
XP 002883385.1 *A. lyrata* subsp. *Lyrata*	-	-	-	-	-	-	-	-	-	-	-	-	-	-	-	-	D	T	-	-	-	R	G	K	H
I3SXY5 *L. japonicus*	-	-	-	-	-	-	-	-	-	-	-	-	-	-	-	A	D	T	-	-	-	R	G	K	H
Bra023782 *B. rapa*	-	-	-	-	-	-	-	-	-	-	-	-	-	-	-	-	D	T	-	-	-	R	G	K	H
ACX43275.1 *B. napus*	-	-	-	-	-	-	-	-	-	-	-	-	-	-	-	-	D	T	-	-	-	R	G	K	H
Thhalv10021823m *T. halophila*	-	-	-	-	-	-	-	-	-	-	-	-	-	-	-	-	D	T	-	-	-	R	G	K	H
Phvul.007G111000.1 *P. vulgaris*	-	-	-	-	-	-	-	-	-	-	-	-	-	-	-	A	D	T	-	-	-	R	G	K	H
FF545146.1 *V. unguiculata*	-	-	-	-	-	-	-	-	-	-	-	-	-	-	-	A	D	T	-	-	-	R	G	K	H
I3T376 *L. japonicus*	-	-	-	-	-	-	-	-	-	-	-	-	-	-	-	A	D	T	-	-	-	R	G	K	H
Carubv10014985m *C. rubella*	-	-	-	-	-	-	-	-	-	-	-	-	-	-	-	-	D	T	-	-	-	R	G	K	H
NP 850746.1 *A. thalina* AGG2	-	-	-	-	-	-	-	-	-	-	-	-	-	-	-	V	D	T	-	-	-	R	G	K	H
*Pa*GG1	-	-	-	-	-	-	-	-	-	-	-	-	-	-	-	T	D	V	-	-	-	G	G	K	H
ADE76615.1 *P. sitchensis*	-	-	-	-	-	-	-	-	-	-	-	-	-	-	-	T	D	V	-	-	-	G	G	K	H
EX389861.1 *P. glauca*	-	-	-	-	-	-	-	-	-	-	-	-	-	-	-	T	D	V	-	-	-	G	G	K	H
CO158867.1 *P. taeda*	-	-	-	-	-	-	-	-	-	-	-	-	-	-	-	M	D	V	-	-	-	G	G	K	H
BX254993.1/BX680269.1 *P. pinaster*	-	-	-	-	-	-	-	-	-	-	-	-	-	-	-	M	D	V	-	-	-	G	G	K	H
GT252854.1 *P. contorta*	-	-	-	-	-	-	-	-	-	-	-	-	-	-	-	M	D	V	-	-	-	G	G	K	H
Phvul.001G057600.1 *P. vulgaris*	-	-	-	-	-	-	-	-	-	-	-	-	-	-	-	G	-	F	-	-	-	I	G	K	H
I3SQN8 *L. japonicus*	-	-	-	-	-	-	-	-	-	-	-	-	-	-	-	A	-	F	-	-	-	Y	G	K	H
*Pa*GG2	-	-	-	-	-	-	-	-	-	-	-	-	-	-	-	N	-	L	-	-	-	R	G	I	Q
DR093846 *P. taeda*	-	-	-	-	-	-	-	-	-	-	-	-	-	-	-	-	D	L	-	-	-	R	G	I	N
GT123649.1 *P. sitchensis*	-	-	-	-	-	-	-	-	-	-	-	-	-	-	-	N	-	L	-	-	-	R	G	I	Q
BT117176.1 *P. glauca*	-	-	-	-	-	-	-	-	-	-	-	-	-	-	-	N	-	L	-	-	-	R	G	I	Q

f

ABK26455.1 *P. sitchensis*	R	K	Q	V	E	L	N	R	L	T	K	E	I	S	L	L	E	E	E	L	K	T	L	E	G
*Pa*GG3	R	K	Q	V	E	L	N	R	L	T	K	E	I	S	L	L	E	E	E	L	K	T	L	E	G
BT111616.1 *P. glauca*	R	K	R	V	E	L	N	R	L	T	K	E	I	S	L	L	E	E	E	L	K	T	L	E	G
DT638145.1 *P. taeda*	R	K	Q	V	E	L	N	R	L	T	K	E	I	S	L	L	E	E	E	L	K	T	L	E	G
Pp1s22 182V6.1 *P. patens*	R	D	L	H	Q	L	N	T	-	E	I	Q	-	-	L	L	Q	E	E	L	N	S	L	D	D
Pp1s39 119V6.2*P. Patens*	R	K	Q	T	E	L	Q	H	L	N	N	E	I	Q	L	L	Q	E	E	L	E	V	L	N	N
Medtr8g021170.1 *M. truncatula*	K	Q	M	V	K	I	Q	V	L	E	K	E	I	G	L	L	Q	E	E	L	K	S	L	E	G
Phvul.003G130200.1 *P. vulgaris*	R	E	T	A	R	V	H	M	L	E	R	E	I	T	F	L	E	E	E	L	K	S	V	E	G
Medtr4g125190.1 *M. truncatula*	R	E	M	A	K	V	Q	M	L	E	R	E	I	G	F	L	E	E	E	L	K	S	M	E	G
Medtr2g042200.1 *M. truncatula*	R	E	M	A	K	V	Q	M	L	E	R	E	I	S	F	L	E	E	E	L	K	S	S	E	G
Carubv10002620m *C. rubella*	R	E	A	A	R	V	Q	M	L	E	R	E	I	G	F	L	E	G	E	I	K	F	V	E	G
Thhalv10014548m *T. halophila*	R	E	A	A	R	V	Q	M	L	E	R	E	I	G	F	L	E	G	E	I	K	F	I	E	G
XP 002874008.1 *A. lyrata* subsp. *Lyrata*	R	E	A	A	R	V	Q	M	L	S	R	E	I	G	F	L	E	G	E	I	K	F	T	E	G
AT5G20635.1 *A. thaliana* AGG3	R	E	A	A	R	V	Q	M	L	E	R	E	I	G	F	L	E	G	E	I	K	F	I	E	G
ADE77369.1 *P. sitchensis*	R	K	Q	A	E	L	D	Q	L	N	R	E	I	S	S	L	E	E	E	L	I	S	L	E	G
*Pa*GG4	R	K	Q	A	E	L	D	Q	L	N	R	E	I	S	S	L	E	E	E	L	I	S	L	E	G
Phvul.007G175700.1 *P. vulgaris*	R	I	L	A	E	L	K	R	L	D	Q	D	S	K	F	L	Q	E	E	L	E	E	L	E	K
G7ICP9 *M. truncatula*	R	I	L	A	E	L	K	R	L	Q	Q	D	T	K	F	L	Q	E	E	L	D	E	L	E	K
Bra007741 *B. rapa*	R	I	L	A	E	L	G	R	V	E	Q	E	V	I	F	L	E	K	E	L	E	E	L	G	Q
EE552948.1 *B. napus*	R	I	L	A	E	L	G	R	V	E	E	E	V	R	F	L	E	K	E	L	E	E	L	G	Q
FD578839.1 *R. sativus*	R	I	L	A	E	L	G	R	V	E	Q	E	V	K	F	L	E	K	E	L	E	E	L	G	Q
Thhalv10006334m *T. halophila*	R	I	L	A	E	L	G	R	V	E	Q	E	V	R	F	L	E	K	E	L	E	E	L	G	H
Carubv10018305m *C. rubella*	R	I	L	A	D	L	A	R	V	E	Q	E	L	V	F	L	E	K	E	L	V	E	V	E	Q
XP 002876721.1 *A. lyrata* subsp. *Lyrata*	R	I	L	A	E	L	A	R	V	E	Q	E	V	V	F	L	E	K	E	L	E	E	V	E	S
NP 567147.1 *A. thaliana* AGG1	R	I	L	A	E	L	A	R	V	E	Q	E	V	A	F	L	E	K	E	L	K	E	V	E	N
FF393368.1 *V. unguiculata*	R	I	L	A	E	L	K	R	L	D	Q	D	S	K	F	L	Q	E	E	L	E	E	L	E	K
XP 002883385.1 *A. lyrata* subsp. *Lyrata*	R	I	Q	A	E	L	K	R	L	E	Q	E	A	R	F	L	E	E	E	L	E	Q	L	E	K
I3SXY5 *L. japonicus*	R	V	H	A	E	L	K	R	T	E	Q	E	A	K	F	L	E	E	E	L	E	Q	L	E	K
Bra023782 *B. rapa*	R	I	H	A	Q	L	N	R	L	Q	Q	E	A	R	F	L	E	E	E	L	E	Q	L	E	K
ACX43275.1 *B. napus*	R	I	H	A	E	L	K	R	L	E	Q	E	A	R	F	L	E	E	E	L	E	Q	L	E	K
Thhalv10021823m *T. halophila*	R	I	Q	A	E	L	K	R	L	E	Q	E	T	R	F	L	E	E	E	L	E	Q	L	E	K
Phvul.007G111000.1 *P. vulgaris*	R	I	H	A	E	L	K	R	L	E	Q	E	A	R	F	L	E	E	E	L	E	K	L	E	R
FF545146.1 *V. unguiculata*	R	I	H	A	E	L	K	R	L	E	Q	E	A	R	F	L	E	E	E	L	E	K	L	E	R
I3T376 *L. japonicus*	R	I	H	A	E	L	K	R	L	E	Q	E	A	R	F	L	E	E	E	L	E	Q	L	E	R
Carubv10014985m *C. rubella*	R	I	Q	A	E	L	K	R	L	D	Q	E	A	R	F	L	E	E	E	L	E	Q	L	E	R
NP 850746.1 *A. thalina* AGG2	R	I	Q	A	E	L	K	R	L	E	Q	E	A	R	F	L	E	E	E	L	E	Q	L	E	K
*Pa*GG1	R	K	L	A	E	L	H	R	L	N	Q	E	I	R	F	L	E	E	E	L	E	D	L	D	K
ADE76615.1 *P. sitchensis*	R	K	L	A	E	L	H	R	L	N	Q	E	I	R	F	L	E	E	E	L	E	D	L	D	K
EX389861.1 *P. glauca*	R	K	L	A	E	L	H	R	L	N	Q	E	I	R	F	L	E	E	E	L	E	D	L	D	K
CO158867.1 *P. taeda*	R	K	N	A	E	L	Y	R	L	H	Q	E	I	R	Y	L	E	E	E	L	E	D	L	D	K
BX254993.1/BX680269.1 *P. pinaster*	R	K	N	A	E	L	Y	R	L	Q	Q	E	I	R	Y	L	E	E	E	L	E	D	L	D	K
GT252854.1 *P. contorta*	R	K	N	A	E	L	Y	R	L	H	Q	E	I	R	Y	L	E	E	E	L	E	D	L	D	K
Phvul.001G057600.1 *P. vulgaris*	R	L	Q	A	A	L	T	H	L	S	N	Q	I	T	I	L	Q	E	E	L	E	K	V	E	T
I3SQN8 *L. japonicus*	R	L	Q	A	A	I	S	Q	L	N	N	Q	I	S	I	M	E	E	E	L	K	Q	L	E	T
*Pa*GG2	H	R	L	S	Q	L	N	H	L	E	Q	Q	I	K	L	L	E	D	E	L	E	E	L	H	N
DR093846 *P. taeda*	H	K	L	A	E	L	N	R	L	D	Q	Q	I	K	D	F	E	E	E	L	K	K	L	D	S
GT123649.1 *P. sitchensis*	H	R	L	S	Q	L	N	H	L	E	Q	Q	I	K	L	L	E	D	E	L	E	E	L	H	N
BT117176.1 *P. glauca*	H	R	L	S	Q	L	N	H	L	E	Q	Q	I	K	L	L	E	D	E	L	E	E	L	H	N

g

ABK26455.1 *P. sitchensis*	L	P	P	S	S	K	C	C	K	G	V	V	E	S	I	E	K	R	P	D	P	L	L	P	F
*Pa*GG3	L	P	P	S	S	K	C	C	K	G	V	V	E	S	I	E	K	R	P	D	P	L	L	P	F
BT111616.1 *P. glauca*	L	P	P	S	S	K	C	C	K	G	V	V	E	S	I	E	K	R	P	D	P	L	L	P	F
DT638145.1 *P. taeda*	L	P	P	A	S	K	C	C	K	G	V	V	E	S	I	E	K	R	P	D	P	L	L	P	F
Pp1s22 182V6.1 *P. patens*	T	P	P	A	S	K	A	C	K	D	L	V	A	F	V	E	S	R	P	D	P	F	I	P	S
Pp1s39 119V6.2*P. Patens*	T	P	L	A	S	K	A	C	K	D	L	I	T	F	V	E	N	L	P	D	P	F	L	S	S
Medtr8g021170.1 *M. truncatula*	L	H	P	A	S	R	C	C	I	E	L	D	A	F	V	E	E	S	T	Q	S	L	N	P	I
Phvul.003G130200.1 *P. vulgaris*	L	Q	P	A	S	R	C	C	K	E	V	A	D	Y	V	M	A	N	P	D	P	L	L	P	-
Medtr4g125190.1 *M. truncatula*	L	Q	P	A	S	K	C	C	K	E	I	A	D	Y	V	V	A	N	S	D	P	L	L	P	-
Medtr2g042200.1 *M. truncatula*	F	Q	P	A	S	K	C	C	K	E	I	A	D	F	V	M	A	N	S	D	P	L	L	P	-
Carubv10002620m *C. rubella*	V	Q	P	A	S	R	C	C	K	E	V	S	D	F	V	A	A	N	S	D	P	M	I	P	A
Thhalv10014548m *T. halophila*	V	Q	P	A	S	R	C	C	K	E	V	S	D	F	V	V	A	N	S	D	P	L	I	P	A
XP 002874008.1 *A. lyrata* subsp. *Lyrata*	V	Q	P	A	S	R	C	C	K	E	V	S	D	F	V	V	A	N	S	D	P	L	I	P	A
AT5G20635.1 *A. thaliana* AGG3	V	Q	P	A	S	R	C	I	K	E	V	S	D	F	V	V	A	N	S	D	P	L	I	P	A
ADE77369.1 *P. sitchensis*	L	P	P	A	S	I	C	C	K	E	V	E	E	S	V	N	A	R	S	D	P	L	L	P	P
*Pa*GG4	L	P	P	A	S	I	C	C	K	E	V	E	E	S	V	N	A	R	S	D	P	L	L	P	R
Phvul.007G175700.1 *P. vulgaris*	T	E	N	V	S	T	I	C	M	D	L	L	Q	S	M	E	S	R	P	D	P	L	L	P	E
G7ICP9 *M. truncatula*	T	E	N	V	S	A	I	C	K	E	L	L	Q	N	M	D	S	R	P	D	P	L	I	P	E
Bra007741 *B. rapa*	T	D	I	V	S	A	V	C	E	E	L	L	C	L	I	E	K	A	P	D	P	L	L	P	L
EE552948.1 *B. napus*	T	D	T	V	P	T	V	C	E	E	L	L	C	L	I	E	K	A	P	D	P	L	L	P	L
FD578839.1 *R. sativus*	T	D	I	V	S	T	V	C	E	E	L	F	C	V	I	E	K	A	P	D	P	L	L	P	L
Thhalv10006334m *T. halophila*	T	D	I	V	S	N	V	C	E	E	L	L	C	V	I	E	K	A	P	D	P	L	L	P	L
Carubv10018305m *C. rubella*	T	E	I	V	S	A	V	C	E	E	L	L	C	V	I	E	K	G	P	D	P	L	L	P	L
XP 002876721.1 *A. lyrata* subsp. *Lyrata*	T	D	I	V	S	T	V	C	E	E	L	L	C	V	V	E	K	G	P	D	P	L	L	P	L
NP 567147.1 *A. thaliana* AGG1	T	D	I	V	S	T	V	C	E	E	L	L	S	V	I	E	K	G	P	D	P	L	L	P	L
FF393368.1 *V. unguiculata*	T	E	N	V	S	T	I	C	T	D	L	L	Q	S	M	E	-	-	S	R	P	-	-	-	-
XP 002883385.1 *A. lyrata* subsp. *Lyrata*	M	D	N	A	S	A	S	C	K	E	F	L	D	S	V	E	S	K	P	D	P	L	L	P	E
I3SXY5 *L. japonicus*	M	E	K	A	S	T	S	C	K	E	M	I	S	N	V	E	A	R	P	D	P	L	L	P	L
Bra023782 *B. rapa*	M	D	K	A	S	A	S	C	K	E	F	L	D	S	V	D	S	K	P	D	P	L	L	P	L
ACX43275.1 *B. napus*	M	D	T	A	S	A	S	C	K	E	F	L	D	S	V	E	S	K	P	D	P	L	L	P	E
Thhalv10021823m *T. halophila*	M	D	N	A	S	A	C	C	K	E	F	L	D	S	V	D	S	N	P	D	P	L	L	P	E
Phvul.007G111000.1 *P. vulgaris*	M	E	K	A	S	T	A	C	K	G	M	L	S	N	V	E	T	K	P	D	P	L	L	P	-
FF545146.1 *V. unguiculata*	M	E	K	A	S	T	A	C	K	G	L	L	S	N	V	E	T	K	P	D	P	L	L	P	-
I3T376 *L. japonicus*	M	E	K	A	S	T	S	C	K	E	M	L	S	N	V	E	T	R	P	D	P	L	L	P	-
Carubv10014985m *C. rubella*	M	D	N	V	S	S	S	C	K	E	F	L	D	S	V	D	S	K	P	D	P	L	L	P	E
NP 850746.1 *A. thalina* AGG2	M	D	N	A	S	A	S	C	K	E	F	L	D	S	V	D	S	K	P	D	P	L	L	P	E
*Pa*GG1	I	D	K	A	T	S	A	C	K	E	M	L	L	I	I	E	N	T	P	D	P	L	L	S	V
ADE76615.1 *P. sitchensis*	I	D	K	A	T	S	A	C	K	E	M	L	L	I	I	E	N	T	P	D	P	L	L	S	V
EX389861.1 *P. glauca*	I	D	K	A	T	S	A	C	K	E	M	L	L	I	I	E	N	T	P	D	P	L	L	S	V
CO158867.1 *P. taeda*	I	D	K	A	T	S	A	C	K	E	M	L	L	I	I	E	N	T	P	D	P	L	L	S	V
BX254993.1/BX680269.1 *P. pinaster*	I	D	K	A	T	S	A	C	K	E	M	L	L	L	I	E	N	T	P	D	P	L	L	S	V
GT252854.1 *P. contorta*	I	D	K	A	T	S	A	C	K	E	M	L	L	I	I	E	N	T	P	D	P	L	L	S	V
Phvul.001G057600.1 *P. vulgaris*	I	G	E	S	S	T	V	C	K	D	L	I	S	S	V	E	S	T	A	D	P	L	L	P	C
I3SQN8 *L. japonicus*	I	G	E	S	S	I	V	C	K	D	V	I	S	S	V	E	S	I	P	D	P	L	L	P	F
*Pa*GG2	T	D	R	A	S	T	V	C	R	D	V	L	M	T	V	D	S	R	P	D	A	F	L	P	-
DR093846 *P. taeda*	T	D	R	A	S	T	V	C	G	E	V	L	V	T	I	D	S	R	P	D	A	L	L	P	-
GT123649.1 *P. sitchensis*	T	D	R	A	S	T	V	C	R	D	V	L	M	T	V	D	S	R	P	D	A	F	L	P	S
BT117176.1 *P. glauca*	T	D	R	A	S	T	V	R	R	D	V	L	M	T	V	D	S	R	P	D	A	F	L	P	S

h

ABK26455.1 *P. sitchensis*	V	T	R	-	-	-	-	-	-	-	-	-	-	-	-	-	-	-	-	-	-	-	-	-	-
*Pa*GG3	V	T	R	-	-	-	-	-	-	-	-	-	-	-	-	-	-	-	-	-	-	-	-	-	-
BT111616.1 *P. glauca*	V	T	R	-	-	-	-	-	-	-	-	-	-	-	-	-	-	-	-	-	-	-	-	-	-
DT638145.1 *P. taeda*	V	S	R	-	-	-	-	-	-	-	-	-	-	-	-	-	-	-	-	-	-	-	-	-	-
Pp1s22 182V6.1 *P. patens*	S	D	G	I	-	-	-	-	-	-	-	-	-	-	-	-	-	-	-	-	-	-	-	-	-
Pp1s39 119V6.2*P. Patens*	S	D	G	V	-	-	-	-	-	-	-	-	-	-	-	-	-	-	-	-	-	-	-	-	-
Medtr8g021170.1 *M. truncatula*	T	S	-	-	-	-	-	-	-	-	-	-	-	-	-	-	-	-	-	-	-	-	-	-	-
Phvul.003G130200.1 *P. vulgaris*	T	T	K	K	N	R	R	S	C	R	F	W	K	W	-	-	-	-	L	C	-	-	-	-	-
Medtr4g125190.1 *M. truncatula*	S	N	K	K	N	R	R	S	C	R	F	W	K	W	L	F	-	-	M	C	S	R	L	P	-
Medtr2g042200.1 *M. truncatula*	T	T	K	K	N	R	K	T	C	R	L	W	K	W	L	W	Y	D	F	Y	-	F	L	T	R
Carubv10002620m *C. rubella*	Q	-	R	K	S	R	R	S	C	R	F	W	K	W	-	-	-	-	L	C	-	-	-	-	-
Thhalv10014548m *T. halophila*	S	-	-	-	-	-	-	-	-	-	-	-	-	-	-	-	-	-	-	-	-	-	-	-	-
XP 002874008.1 *A. lyrata* subsp. *Lyrata*	Q	-	R	K	S	R	R	S	C	R	F	W	K	W	-	-	-	-	L	C	-	-	-	-	-
AT5G20635.1 *A. thaliana* AGG3	Q	-	R	K	S	R	R	S	F	R	F	W	K	W	-	-	-	-	L	C	-	-	-	-	-
ADE77369.1 *P. sitchensis*	-	N	K	D	-	-	-	-	-	-	-	-	-	-	-	-	-	-	-	-	-	-	-	-	-
*Pa*GG4	-	N	K	D	-	-	-	-	-	-	-	-	-	-	-	-	-	-	-	-	-	-	-	-	-
Phvul.007G175700.1 *P. vulgaris*	V	H	-	-	-	-	-	-	-	-	-	-	-	-	-	-	-	-	-	-	-	-	-	-	-
G7ICP9 *M. truncatula*	V	H	-	-	-	-	-	-	-	-	-	-	-	-	-	-	-	-	-	-	-	-	-	-	-
Bra007741 *B. rapa*	T	N	-	-	-	-	-	-	-	-	-	-	-	-	-	-	-	-	-	-	-	-	-	-	-
EE552948.1 *B. napus*	T	N	-	-	-	-	-	-	-	-	-	-	-	-	-	-	-	-	-	-	-	-	-	-	-
FD578839.1 *R. sativus*	T	-	K	-	-	-	-	-	-	-	-	-	-	-	-	-	-	-	-	-	-	-	-	-	-
Thhalv10006334m *T. halophila*	T	N	-	-	-	-	-	-	-	-	-	-	-	-	-	-	-	-	-	-	-	-	-	-	-
Carubv10018305m *C. rubella*	T	N	-	-	-	-	-	-	-	-	-	-	-	-	-	-	-	-	-	-	-	-	-	-	-
XP 002876721.1 *A. lyrata* subsp. *Lyrata*	T	N	-	-	-	-	-	-	-	-	-	-	-	-	-	-	-	-	-	-	-	-	-	-	-
NP 567147.1 *A. thaliana* AGG1	T	N	-	-	-	-	-	-	-	-	-	-	-	-	-	-	-	-	-	-	-	-	-	-	-
FF393368.1 *V. unguiculata*	-	-	-	-	-	-	-	-	-	-	-	-	-	-	-	-	-	-	-	-	-	-	-	-	-
XP 002883385.1 *A. lyrata* subsp. *Lyrata*	T	T	-	-	-	-	-	-	-	-	-	-	-	-	-	-	-	-	-	-	-	-	-	-	-
I3SXY5 *L. japonicus*	T	-	A	-	-	-	-	-	-	-	-	-	-	-	-	-	-	-	-	-	-	-	-	-	-
Bra023782 *B. rapa*	T	-	I	-	-	-	-	-	-	-	-	-	-	-	-	-	-	-	-	-	-	-	-	-	-
ACX43275.1 *B. napus*	T	-	I	-	-	-	-	-	-	-	-	-	-	-	-	-	-	-	-	-	-	-	-	-	-
Thhalv10021823m *T. halophila*	T	-	M	-	-	-	-	-	-	-	-	-	-	-	-	-	-	-	-	-	-	-	-	-	-
Phvul.007G111000.1 *P. vulgaris*	S	T	I	-	-	-	-	-	-	-	-	-	-	-	-	-	-	-	-	-	-	-	-	-	-
FF545146.1 *V. unguiculata*	S	T	I	-	-	-	-	-	-	-	-	-	-	-	-	-	-	-	-	-	-	-	-	-	-
I3T376 *L. japonicus*	S	T	V	-	-	-	-	-	-	-	-	-	-	-	-	-	-	-	-	-	-	-	-	-	-
Carubv10014985m *C. rubella*	T	T	-	-	-	-	-	-	-	-	-	-	-	-	-	-	-	-	-	-	-	-	-	-	-
NP 850746.1 *A. thalina* AGG2	T	T	-	-	-	-	-	-	-	-	-	-	-	-	-	-	-	-	-	-	-	-	-	-	-
*Pa*GG1	T	-	K	-	-	-	-	-	-	-	-	-	-	-	-	-	-	-	-	-	-	-	-	-	-
ADE76615.1 *P. sitchensis*	T	-	K	-	-	-	-	-	-	-	-	-	-	-	-	-	-	-	-	-	-	-	-	-	-
EX389861.1 *P. glauca*	T	-	K	-	-	-	-	-	-	-	-	-	-	-	-	-	-	-	-	-	-	-	-	-	-
CO158867.1 *P. taeda*	T	-	K	-	-	-	-	-	-	-	-	-	-	-	-	-	-	-	-	-	-	-	-	-	-
BX254993.1/BX680269.1 *P. pinaster*	T	-	K	-	-	-	-	-	-	-	-	-	-	-	-	-	-	-	-	-	-	-	-	-	-
GT252854.1 *P. contorta*	T	-	K	-	-	-	-	-	-	-	-	-	-	-	-	-	-	-	-	-	-	-	-	-	-
Phvul.001G057600.1 *P. vulgaris*	T	-	K	-	-	-	-	-	-	-	-	-	-	-	-	-	-	-	-	-	-	-	-	-	-
I3SQN8 *L. japonicus*	T	-	K	-	-	-	-	-	-	-	-	-	-	-	-	-	-	-	-	-	-	-	-	-	-
*Pa*GG2	S	T	I	-	-	-	-	-	-	-	-	-	-	-	-	-	-	-	-	-	-	-	-	-	-
DR093846 *P. taeda*	T	T	G	-	-	-	-	-	-	-	-	-	-	-	-	-	-	-	-	-	-	-	-	-	-
GT123649.1 *P. sitchensis*	T	T	-	-	-	-	-	-	-	-	-	-	-	-	-	-	-	-	-	-	-	-	-	-	-
BT117176.1 *P. glauca*	-	T	I	-	-	-	-	-	-	-	-	-	-	-	-	-	-	-	-	-	-	-	-	-	-

i

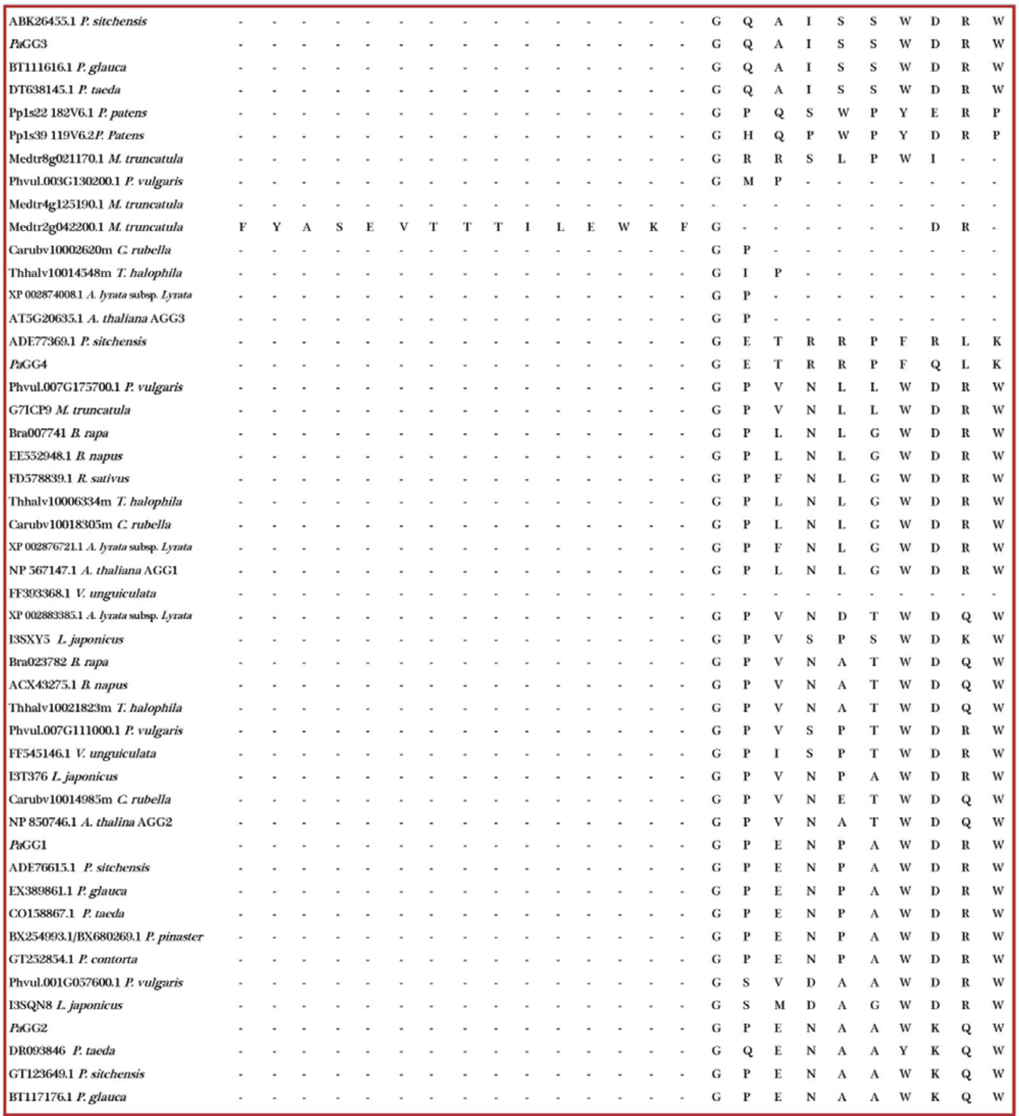

j

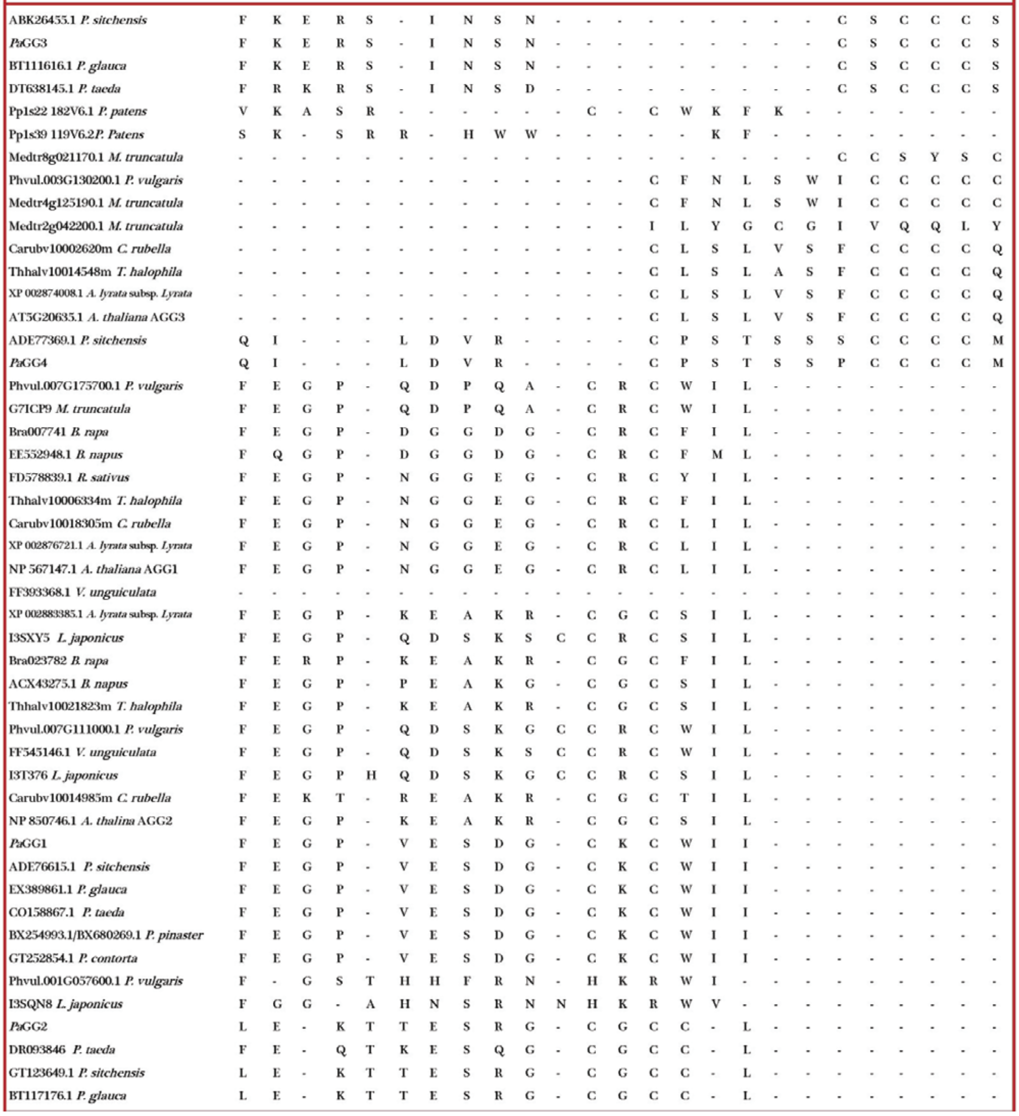

k

ABK26455.1 *P. sitchensis*	C	K	-	-	-	-	-	H	V	K	I	W	K	F	S	Y	-	-	W	K	R	-	-	-	-
*Pa*GG3	C	K	-	-	-	-	-	H	V	K	I	W	K	F	S	Y	-	-	W	K	R	-	-	-	-
BT111616.1 *P. glauca*	C	K	-	-	-	-	-	H	V	K	I	W	K	F	S	Y	-	-	W	K	R	-	-	-	-
DT638145.1 *P. taeda*	C	K	-	-	-	-	-	H	V	K	I	W	K	F	S	Y	-	-	W	K	R	-	-	-	-
Pp1s22 182V6.1 *P. patens*	-	-	-	-	-	-	-	-	-	-	-	-	-	-	-	-	-	-	-	-	-	-	-	-	-
Pp1s39 119V6.2*P. Patens*	-	-	-	-	-	-	-	-	-	-	-	-	-	-	-	-	-	-	-	-	-	-	-	-	-
Medtr8g021170.1 *M. truncatula*	F	P	-	-	-	-	-	H	K	K	I	A	K	G	C	C	S	C	C	S	S	S	N	S	R
Phvul.003G130200.1 *P. vulgaris*	C	C	E	G	L	S	L	P	L	K	L	P	R	-	C	C	G	D	C	K	P	C	S	C	-
Medtr4g125190.1 *M. truncatula*	C	C	D	G	L	S	V	H	L	K	L	P	S	-	C	C	S	D	C	K	P	C	S	C	C
Medtr2g042200.1 *M. truncatula*	S	P	-	-	-	-	-	-	-	-	-	-	-	-	Y	T	C	R	C	C	D	C	F	S	G
Carubv10002620m *C. rubella*	S	K	C	S	C	-	-	H	L	K	K	P	K	-	-	C	C	S	C	T	S	C	S	C	I
Thhalv10014548m *T. halophila*	S	E	C	S	C	-	-	H	L	R	K	P	K	-	-	C	C	N	C	T	G	C	S	C	I
XP 002874008.1 *A. lyrata* subsp. *Lyrata*	S	K	C	S	C	-	-	H	L	R	K	P	K	-	-	C	C	N	C	T	S	C	S	C	I
AT5G20635.1 *A. thaliana* AGG3	S	K	C	S	C	-	-	H	L	R	K	P	K	-	-	C	C	N	C	T	S	C	S	C	I
ADE77369.1 *P. sitchensis*	R	C	K	V	Q	-	-	S	D	A	S	-	-	-	-	C	C	S	C	N	-	-	-	-	-
*Pa*GG4	R	C	K	V	Q	-	-	S	D	A	S	-	-	-	-	C	C	S	C	N	-	-	-	-	-
Phvul.007G175700.1 *P. vulgaris*	-	-	-	-	-	-	-	-	-	-	-	-	-	-	-	-	-	-	-	-	-	-	-	-	-
G7ICP9 *M. truncatula*	-	-	-	-	-	-	-	-	-	-	-	-	-	-	-	-	-	-	-	-	-	-	-	-	-
Bra007741 *B. rapa*	-	-	-	-	-	-	-	-	-	-	-	-	-	-	-	-	-	-	-	-	-	-	-	-	-
EE552948.1 *B. napus*	-	-	-	-	-	-	-	-	-	-	-	-	-	-	-	-	-	-	-	-	-	-	-	-	-
FD578839.1 *R. sativus*	-	-	-	-	-	-	-	-	-	-	-	-	-	-	-	-	-	-	-	-	-	-	-	-	-
Thhalv10006334m *T. halophila*	-	-	-	-	-	-	-	-	-	-	-	-	-	-	-	-	-	-	-	-	-	-	-	-	-
Carubv10018305m *C. rubella*	-	-	-	-	-	-	-	-	-	-	-	-	-	-	-	-	-	-	-	-	-	-	-	-	-
XP 002876721.1 *A. lyrata* subsp. *Lyrata*	-	-	-	-	-	-	-	-	-	-	-	-	-	-	-	-	-	-	-	-	-	-	-	-	-
NP 567147.1 *A. thaliana* AGG1	-	-	-	-	-	-	-	-	-	-	-	-	-	-	-	-	-	-	-	-	-	-	-	-	-
FF393368.1 *V. unguiculata*	-	-	-	-	-	-	-	-	-	-	-	-	-	-	-	-	-	-	-	-	-	-	-	-	-
XP 002883385.1 *A. lyrata* subsp. *Lyrata*	-	-	-	-	-	-	-	-	-	-	-	-	-	-	-	-	-	-	-	-	-	-	-	-	-
I3SXY5 *L. japonicus*	-	-	-	-	-	-	-	-	-	-	-	-	-	-	-	-	-	-	-	-	-	-	-	-	-
Bra023782 *B. rapa*	-	-	-	-	-	-	-	-	-	-	-	-	-	-	-	-	-	-	-	-	-	-	-	-	-
ACX43275.1 *B. napus*	-	-	-	-	-	-	-	-	-	-	-	-	-	-	-	-	-	-	-	-	-	-	-	-	-
Thhalv10021823m *T. halophila*	-	-	-	-	-	-	-	-	-	-	-	-	-	-	-	-	-	-	-	-	-	-	-	-	-
Phvul.007G111000.1 *P. vulgaris*	-	-	-	-	-	-	-	-	-	-	-	-	-	-	-	-	-	-	-	-	-	-	-	-	-
FF545146.1 *V. unguiculata*	-	-	-	-	-	-	-	-	-	-	-	-	-	-	-	-	-	-	-	-	-	-	-	-	-
I3T376 *L. japonicus*	-	-	-	-	-	-	-	-	-	-	-	-	-	-	-	-	-	-	-	-	-	-	-	-	-
Carubv10014985m *C. rubella*	-	-	-	-	-	-	-	-	-	-	-	-	-	-	-	-	-	-	-	-	-	-	-	-	-
NP 850746.1 *A. thalina* AGG2	-	-	-	-	-	-	-	-	-	-	-	-	-	-	-	-	-	-	-	-	-	-	-	-	-
*Pa*GG1	-	-	-	-	-	-	-	-	-	-	-	-	-	-	-	-	-	-	-	-	-	-	-	-	-
ADE76615.1 *P. sitchensis*	-	-	-	-	-	-	-	-	-	-	-	-	-	-	-	-	-	-	-	-	-	-	-	-	-
EX389861.1 *P. glauca*	-	-	-	-	-	-	-	-	-	-	-	-	-	-	-	-	-	-	-	-	-	-	-	-	-
CO158867.1 *P. taeda*	-	-	-	-	-	-	-	-	-	-	-	-	-	-	-	-	-	-	-	-	-	-	-	-	-
BX254993.1/BX680269.1 *P. pinaster*	-	-	-	-	-	-	-	-	-	-	-	-	-	-	-	-	-	-	-	-	-	-	-	-	-
GT252854.1 *P. contorta*	-	-	-	-	-	-	-	-	-	-	-	-	-	-	-	-	-	-	-	-	-	-	-	-	-
Phvul.001G057600.1 *P. vulgaris*	-	-	-	-	-	-	-	-	-	-	-	-	-	-	-	-	-	-	-	-	-	-	-	-	-
I3SQN8 *L. japonicus*	-	-	-	-	-	-	-	-	-	-	-	-	-	-	-	-	-	-	-	-	-	-	-	-	-
*Pa*GG2	-	-	-	-	-	-	-	-	-	-	-	-	-	-	-	-	-	-	-	-	-	-	-	-	-
DR093846 *P. taeda*	-	-	-	-	-	-	-	-	-	-	-	-	-	-	-	-	-	-	-	-	-	-	-	-	-
GT123649.1 *P. sitchensis*	-	-	-	-	-	-	-	-	-	-	-	-	-	-	-	-	-	-	-	-	-	-	-	-	-
BT117176.1 *P. glauca*	-	-	-	-	-	-	-	-	-	-	-	-	-	-	-	-	-	-	-	-	-	-	-	-	-

l

ABK26455.1 *P. sitchensis*	-	-	-	-	-	F	I	C	W	K	L	T	F	R	K	A	C	C	V	F	-	P	R	F	S
*Pa*GG3	-	-	-	-	-	F	L	C	W	K	L	T	F	R	K	A	C	C	V	F	-	P	R	F	S
BT111616.1 *P. glauca*	-	-	-	-	-	F	I	C	W	K	L	T	F	R	K	A	C	C	V	F	-	P	R	F	S
DT638145.1 *P. taeda*	-	-	-	-	-	-	-	-	-	-	-	-	-	-	-	-	-	-	-	-	-	-	-	-	-
Pp1s22 182V6.1 *P. patens*	-	-	-	-	-	-	-	-	-	-	-	-	-	-	-	-	-	-	-	-	-	-	-	-	-
Pp1s39 119V6.2*P. Patens*	-	-	-	-	-	-	-	-	-	-	-	-	-	-	-	-	-	-	-	-	-	-	-	-	-
Medtr8g021170.1 *M. truncatula*	C	-	-	-	-	F	G	N	N	C	-	-	-	-	-	-	-	-	-	-	-	-	-	-	-
Phvul.003G130200.1 *P. vulgaris*	S	C	S	C	-	L	P	S	I	K	C	C	S	L	P	K	W	S	C	C	C	S	C	P	K
Medtr4g125190.1 *M. truncatula*	S	C	S	N	C	F	P	S	F	T	C	-	S	L	P	K	W	N	C	C	C	C	-	-	F
Medtr2g042200.1 *M. truncatula*	C	C	N	C	K	Q	N	C	K	C	S	S	C	L	S	S	T	K	C	S	L	S	N	W	C
Carubv10002620m *C. rubella*	G	S	K	C	C	D	G	S	C	C	S	N	I	-	-	-	-	C	C	C	-	P	R	P	S
Thhalv10014548m *T. halophila*	G	S	K	C	C	D	G	S	C	C	S	D	I	-	-	-	-	C	C	C	-	P	R	P	S
XP 002874008.1 *A. lyrata* subsp. *Lyrata*	G	S	K	C	C	D	G	S	C	C	S	N	I	-	-	-	-	C	C	C	-	P	R	P	S
AT5G20635.1 *A. thaliana* AGG3	G	S	K	C	C	D	G	S	C	C	S	N	I	-	-	-	-	C	C	C	-	P	R	L	S
ADE77369.1 *P. sitchensis*	-	-	-	-	-	-	-	-	-	-	-	-	F	L	K	K	W	R	C	S	C	W	K	M	S
*Pa*GG4	-	-	-	-	-	-	-	-	-	-	-	-	F	L	K	K	W	R	C	S	C	W	K	M	S
Phvul.007G175700.1 *P. vulgaris*	-	-	-	-	-	-	-	-	-	-	-	-	-	-	-	-	-	-	-	-	-	-	-	-	-
G7ICP9 *M. truncatula*	-	-	-	-	-	-	-	-	-	-	-	-	-	-	-	-	-	-	-	-	-	-	-	-	-
Bra007741 *B. rapa*	-	-	-	-	-	-	-	-	-	-	-	-	-	-	-	-	-	-	-	-	-	-	-	-	-
EE552948.1 *B. napus*	-	-	-	-	-	-	-	-	-	-	-	-	-	-	-	-	-	-	-	-	-	-	-	-	-
FD578839.1 *R. sativus*	-	-	-	-	-	-	-	-	-	-	-	-	-	-	-	-	-	-	-	-	-	-	-	-	-
Thhalv10006334m *T. halophila*	-	-	-	-	-	-	-	-	-	-	-	-	-	-	-	-	-	-	-	-	-	-	-	-	-
Carubv10018305m *C. rubella*	-	-	-	-	-	-	-	-	-	-	-	-	-	-	-	-	-	-	-	-	-	-	-	-	-
XP 002876721.1 *A. lyrata* subsp. *Lyrata*	-	-	-	-	-	-	-	-	-	-	-	-	-	-	-	-	-	-	-	-	-	-	-	-	-
NP 567147.1 *A. thaliana* AGG1	-	-	-	-	-	-	-	-	-	-	-	-	-	-	-	-	-	-	-	-	-	-	-	-	-
FF393368.1 *V. unguiculata*	-	-	-	-	-	-	-	-	-	-	-	-	-	-	-	-	-	-	-	-	-	-	-	-	-
XP 002883385.1 *A. lyrata* subsp. *Lyrata*	-	-	-	-	-	-	-	-	-	-	-	-	-	-	-	-	-	-	-	-	-	-	-	-	-
I3SXY5 *L. japonicus*	-	-	-	-	-	-	-	-	-	-	-	-	-	-	-	-	-	-	-	-	-	-	-	-	-
Bra023782 *B. rapa*	-	-	-	-	-	-	-	-	-	-	-	-	-	-	-	-	-	-	-	-	-	-	-	-	-
ACX43275.1 *B. napus*	-	-	-	-	-	-	-	-	-	-	-	-	-	-	-	-	-	-	-	-	-	-	-	-	-
Thhalv10021823m *T. halophila*	-	-	-	-	-	-	-	-	-	-	-	-	-	-	-	-	-	-	-	-	-	-	-	-	-
Phvul.007G111000.1 *P. vulgaris*	-	-	-	-	-	-	-	-	-	-	-	-	-	-	-	-	-	-	-	-	-	-	-	-	-
FF545146.1 *V. unguiculata*	-	-	-	-	-	-	-	-	-	-	-	-	-	-	-	-	-	-	-	-	-	-	-	-	-
I3T376 *L. japonicus*	-	-	-	-	-	-	-	-	-	-	-	-	-	-	-	-	-	-	-	-	-	-	-	-	-
Carubv10014985m *C. rubella*	-	-	-	-	-	-	-	-	-	-	-	-	-	-	-	-	-	-	-	-	-	-	-	-	-
NP 850746.1 *A. thalina* AGG2	-	-	-	-	-	-	-	-	-	-	-	-	-	-	-	-	-	-	-	-	-	-	-	-	-
*Pa*GG1	-	-	-	-	-	-	-	-	-	-	-	-	-	-	-	-	-	-	-	-	-	-	-	-	-
ADE76615.1 *P. sitchensis*	-	-	-	-	-	-	-	-	-	-	-	-	-	-	-	-	-	-	-	-	-	-	-	-	-
EX389861.1 *P. glauca*	-	-	-	-	-	-	-	-	-	-	-	-	-	-	-	-	-	-	-	-	-	-	-	-	-
CO158867.1 *P. taeda*	-	-	-	-	-	-	-	-	-	-	-	-	-	-	-	-	-	-	-	-	-	-	-	-	-
BX254993.1/BX680269.1 *P. pinaster*	-	-	-	-	-	-	-	-	-	-	-	-	-	-	-	-	-	-	-	-	-	-	-	-	-
GT252854.1 *P. contorta*	-	-	-	-	-	-	-	-	-	-	-	-	-	-	-	-	-	-	-	-	-	-	-	-	-
Phvul.001G057600.1 *P. vulgaris*	-	-	-	-	-	-	-	-	-	-	-	-	-	-	-	-	-	-	-	-	-	-	-	-	-
I3SQN8 *L. japonicus*	-	-	-	-	-	-	-	-	-	-	-	-	-	-	-	-	-	-	-	-	-	-	-	-	-
*Pa*GG2	-	-	-	-	-	-	-	-	-	-	-	-	-	-	-	-	-	-	-	-	-	-	-	-	-
DR093846 *P. taeda*	-	-	-	-	-	-	-	-	-	-	-	-	-	-	-	-	-	-	-	-	-	-	-	-	-
GT123649.1 *P. sitchensis*	-	-	-	-	-	-	-	-	-	-	-	-	-	-	-	-	-	-	-	-	-	-	-	-	-
BT117176.1 *P. glauca*	-	-	-	-	-	-	-	-	-	-	-	-	-	-	-	-	-	-	-	-	-	-	-	-	-

m

ABK26455.1 *P. sitchensis*	C	H	K	-	-	C	C	C	F	Q	C	S	C	A	G	C	I	K	-	-	C	C	P	L	C
*Pa*GG3	C	Y	K	-	-	C	C	C	F	Q	C	S	C	A	G	C	I	K	-	-	C	C	P	L	C
BT111616.1 *P. glauca*	C	Y	K	-	-	C	C	C	F	Q	C	S	W	A	G	C	I	K	-	-	C	C	P	L	C
DT638145.1 *P. taeda*	-	-	-	-	-	-	-	-	-	-	-	S	S	A	G	S	-	-	-	-	-	-	-	-	-
Pp1s22 182V6.1 *P. patens*	-	-	-	-	-	-	-	-	-	-	-	-	-	-	-	-	-	-	-	-	-	-	-	-	-
Pp1s39 119V6.2*P. Patens*	-	-	-	-	-	-	-	-	-	-	-	-	-	-	-	-	-	-	-	-	-	-	-	-	-
Medtr8g021170.1 *M. truncatula*	-	-	-	-	-	-	-	-	-	-	-	-	-	-	-	-	-	-	-	-	-	-	-	-	-
Phvul.003G130200.1 *P. vulgaris*	S	Y	-	-	-	-	-	F	C	K	E	S	C	G	F	G	N	-	-	-	-	C	C	T	L
Medtr4g125190.1 *M. truncatula*	S	C	P	K	S	N	C	C	K	-	Q	S	L	G	S	G	N	-	-	-	-	C	C	T	F
Medtr2g042200.1 *M. truncatula*	C	C	F	D	K	K	S	H	C	C	K	E	F	C	G	C	N	N	C	C	C	I	L	S	G
Carubv10002620m *C. rubella*	C	P	S	C	P	S	C	P	S	C	S	C	F	R	-	-	-	-	-	-	-	D	C	C	S
Thhalv10014548m *T. halophila*	C	P	S	C	P	S	C	S	S	C	S	C	F	R	G	C	C	C	S	C	P	D	L	S	C
XP 002874008.1 *A. lyrata* subsp. *Lyrata*	C	P	S	C	S	C	-	-	-	-	-	-	F	R	G	C	C	C	S	C	P	D	M	S	C
AT5G20635.1 *A. thaliana* AGG3	C	P	S	C	S	C	-	-	-	-	-	-	F	R	G	C	W	C	S	C	P	D	M	S	C
ADE77369.1 *P. sitchensis*	C	N	S	C	-	C	K	I	P	S	I	S	C	-	G	K	C	S	-	F	L	E	C	S	C
*Pa*GG4	C	N	S	C	-	C	K	I	P	S	I	S	C	-	G	K	C	S	-	F	L	E	C	S	C
Phvul.007G175700.1 *P. vulgaris*	-	-	-	-	-	-	-	-	-	-	-	-	-	-	-	-	-	-	-	-	-	-	-	-	-
G7ICP9 *M. truncatula*	-	-	-	-	-	-	-	-	-	-	-	-	-	-	-	-	-	-	-	-	-	-	-	-	-
Bra007741 *B. rapa*	-	-	-	-	-	-	-	-	-	-	-	-	-	-	-	-	-	-	-	-	-	-	-	-	-
EE552948.1 *B. napus*	-	-	-	-	-	-	-	-	-	-	-	-	-	-	-	-	-	-	-	-	-	-	-	-	-
FD578839.1 *R. sativus*	-	-	-	-	-	-	-	-	-	-	-	-	-	-	-	-	-	-	-	-	-	-	-	-	-
Thhalv10006334m *T. halophila*	-	-	-	-	-	-	-	-	-	-	-	-	-	-	-	-	-	-	-	-	-	-	-	-	-
Carubv10018305m *C. rubella*	-	-	-	-	-	-	-	-	-	-	-	-	-	-	-	-	-	-	-	-	-	-	-	-	-
XP 002876721.1 *A. lyrata* subsp. *Lyrata*	-	-	-	-	-	-	-	-	-	-	-	-	-	-	-	-	-	-	-	-	-	-	-	-	-
NP 567147.1 *A. thaliana* AGG1	-	-	-	-	-	-	-	-	-	-	-	-	-	-	-	-	-	-	-	-	-	-	-	-	-
FF393368.1 *V. unguiculata*	-	-	-	-	-	-	-	-	-	-	-	-	-	-	-	-	-	-	-	-	-	-	-	-	-
XP 002883385.1 *A. lyrata* subsp. *Lyrata*	-	-	-	-	-	-	-	-	-	-	-	-	-	-	-	-	-	-	-	-	-	-	-	-	-
I3SXY5 *L. japonicus*	-	-	-	-	-	-	-	-	-	-	-	-	-	-	-	-	-	-	-	-	-	-	-	-	-
Bra023782 *B. rapa*	-	-	-	-	-	-	-	-	-	-	-	-	-	-	-	-	-	-	-	-	-	-	-	-	-
ACX43275.1 *B. napus*	-	-	-	-	-	-	-	-	-	-	-	-	-	-	-	-	-	-	-	-	-	-	-	-	-
Thhalv10021823m *T. halophila*	-	-	-	-	-	-	-	-	-	-	-	-	-	-	-	-	-	-	-	-	-	-	-	-	-
Phvul.007G111000.1 *P. vulgaris*	-	-	-	-	-	-	-	-	-	-	-	-	-	-	-	-	-	-	-	-	-	-	-	-	-
FF545146.1 *V. unguiculata*	-	-	-	-	-	-	-	-	-	-	-	-	-	-	-	-	-	-	-	-	-	-	-	-	-
I3T376 *L. japonicus*	-	-	-	-	-	-	-	-	-	-	-	-	-	-	-	-	-	-	-	-	-	-	-	-	-
Carubv10014985m *C. rubella*	-	-	-	-	-	-	-	-	-	-	-	-	-	-	-	-	-	-	-	-	-	-	-	-	-
NP 850746.1 *A. thalina* AGG2	-	-	-	-	-	-	-	-	-	-	-	-	-	-	-	-	-	-	-	-	-	-	-	-	-
*Pa*GG1	-	-	-	-	-	-	-	-	-	-	-	-	-	-	-	-	-	-	-	-	-	-	-	-	-
ADE76615.1 *P. sitchensis*	-	-	-	-	-	-	-	-	-	-	-	-	-	-	-	-	-	-	-	-	-	-	-	-	-
EX389861.1 *P. glauca*	-	-	-	-	-	-	-	-	-	-	-	-	-	-	-	-	-	-	-	-	-	-	-	-	-
CO158867.1 *P. taeda*	-	-	-	-	-	-	-	-	-	-	-	-	-	-	-	-	-	-	-	-	-	-	-	-	-
BX254993.1/BX680269.1 *P. pinaster*	-	-	-	-	-	-	-	-	-	-	-	-	-	-	-	-	-	-	-	-	-	-	-	-	-
GT252854.1 *P. contorta*	-	-	-	-	-	-	-	-	-	-	-	-	-	-	-	-	-	-	-	-	-	-	-	-	-
Phvul.001G057600.1 *P. vulgaris*	-	-	-	-	-	-	-	-	-	-	-	-	-	-	-	-	-	-	-	-	-	-	-	-	-
I3SQN8 *L. japonicus*	-	-	-	-	-	-	-	-	-	-	-	-	-	-	-	-	-	-	-	-	-	-	-	-	-
*Pa*GG2	-	-	-	-	-	-	-	-	-	-	-	-	-	-	-	-	-	-	-	-	-	-	-	-	-
DR093846 *P. taeda*	-	-	-	-	-	-	-	-	-	-	-	-	-	-	-	-	-	-	-	-	-	-	-	-	-
GT123649.1 *P. sitchensis*	-	-	-	-	-	-	-	-	-	-	-	-	-	-	-	-	-	-	-	-	-	-	-	-	-
BT117176.1 *P. glauca*	-	-	-	-	-	-	-	-	-	-	-	-	-	-	-	-	-	-	-	-	-	-	-	-	-

n

ABK26455.1 *P. sitchensis*	K	N	K	-	-	-	-	-	-	-	-	-	-	-	-	-	-	-	-	-	-	-	-	-	-
*Pa*GG3	K	N	K	-	-	-	-	-	-	-	-	-	-	-	-	-	-	-	-	-	-	-	-	-	-
BT111616.1 *P. glauca*	K	N	K	-	-	-	-	-	-	-	-	-	-	-	-	-	-	-	-	-	-	-	-	-	-
DT638145.1 *P. taeda*	-	-	-	-	-	-	-	-	-	-	-	-	-	-	-	-	-	-	-	-	-	-	-	-	-
Pp1s22 182V6.1 *P. patens*	-	-	-	-	-	-	-	-	-	-	-	-	-	-	-	-	-	-	-	-	-	-	-	-	-
Pp1s39 119V6.2*P. Patens*	-	-	-	-	-	-	-	-	-	-	-	-	-	-	-	-	-	-	-	-	-	-	-	-	-
Medtr8g021170.1 *M. truncatula*	-	-	-	-	-	-	-	-	-	-	-	-	-	-	-	-	-	-	-	-	-	-	-	-	-
Phvul.003G130200.1 *P. vulgaris*	P	R	S	C	N	-	-	F	G	Y	P	T	C	P	S	C	P	S	C	C	-	-	-	-	-
Medtr4g125190.1 *M. truncatula*	P	T	S	C	N	-	-	F	G	C	P	S	-	-	-	-	-	-	-	-	-	-	-	-	-
Medtr2g042200.1 *M. truncatula*	C	N	F	R	W	P	F	-	P	S	C	C	-	-	-	-	-	-	-	-	-	-	-	-	-
Carubv10002620m *C. rubella*	C	P	D	L	S	C	C	I	P	T	C	F	R	S	C	S	C	A	R	P	S	C	V	S	K
Thhalv10014548m *T. halophila*	C	I	P	T	C	F	R	G	C	T	-	-	-	-	-	-	-	-	R	P	S	C	L	S	K
XP 002874008.1 *A. lyrata* subsp. *Lyrata*	C	I	P	S	C	F	R	N	C	S	C	T	-	-	-	-	-	-	R	P	S	C	L	N	K
AT5G20635.1 *A. thaliana* AGG3	C	I	P	S	C	F	R	S	C	S	C	T	-	-	-	-	-	-	R	P	S	C	L	N	K
ADE77369.1 *P. sitchensis*	-	F	K	C	V	K	C	C	C	P	C	R	R	K	-	-	-	-	-	-	-	-	-	-	-
*Pa*GG4	-	F	K	C	V	K	C	C	C	P	C	R	R	K	-	-	-	-	-	-	-	-	-	-	-
Phvul.007G175700.1 *P. vulgaris*	-	-	-	-	-	-	-	-	-	-	-	-	-	-	-	-	-	-	-	-	-	-	-	-	-
G7ICP9 *M. truncatula*	-	-	-	-	-	-	-	-	-	-	-	-	-	-	-	-	-	-	-	-	-	-	-	-	-
Bra007741 *B. rapa*	-	-	-	-	-	-	-	-	-	-	-	-	-	-	-	-	-	-	-	-	-	-	-	-	-
EE552948.1 *B. napus*	-	-	-	-	-	-	-	-	-	-	-	-	-	-	-	-	-	-	-	-	-	-	-	-	-
FD578839.1 *R. sativus*	-	-	-	-	-	-	-	-	-	-	-	-	-	-	-	-	-	-	-	-	-	-	-	-	-
Thhalv10006334m *T. halophila*	-	-	-	-	-	-	-	-	-	-	-	-	-	-	-	-	-	-	-	-	-	-	-	-	-
Carubv10018305m *C. rubella*	-	-	-	-	-	-	-	-	-	-	-	-	-	-	-	-	-	-	-	-	-	-	-	-	-
XP 002876721.1 *A. lyrata* subsp. *Lyrata*	-	-	-	-	-	-	-	-	-	-	-	-	-	-	-	-	-	-	-	-	-	-	-	-	-
NP 567147.1 *A. thaliana* AGG1	-	-	-	-	-	-	-	-	-	-	-	-	-	-	-	-	-	-	-	-	-	-	-	-	-
FF393368.1 *V. unguiculata*	-	-	-	-	-	-	-	-	-	-	-	-	-	-	-	-	-	-	-	-	-	-	-	-	-
XP 002883385.1 *A. lyrata* subsp. *Lyrata*	-	-	-	-	-	-	-	-	-	-	-	-	-	-	-	-	-	-	-	-	-	-	-	-	-
I3SXY5 *L. japonicus*	-	-	-	-	-	-	-	-	-	-	-	-	-	-	-	-	-	-	-	-	-	-	-	-	-
Bra023782 *B. rapa*	-	-	-	-	-	-	-	-	-	-	-	-	-	-	-	-	-	-	-	-	-	-	-	-	-
ACX43275.1 *B. napus*	-	-	-	-	-	-	-	-	-	-	-	-	-	-	-	-	-	-	-	-	-	-	-	-	-
Thhalv10021823m *T. halophila*	-	-	-	-	-	-	-	-	-	-	-	-	-	-	-	-	-	-	-	-	-	-	-	-	-
Phvul.007G111000.1 *P. vulgaris*	-	-	-	-	-	-	-	-	-	-	-	-	-	-	-	-	-	-	-	-	-	-	-	-	-
FF545146.1 *V. unguiculata*	-	-	-	-	-	-	-	-	-	-	-	-	-	-	-	-	-	-	-	-	-	-	-	-	-
I3T376 *L. japonicus*	-	-	-	-	-	-	-	-	-	-	-	-	-	-	-	-	-	-	-	-	-	-	-	-	-
Carubv10014985m *C. rubella*	-	-	-	-	-	-	-	-	-	-	-	-	-	-	-	-	-	-	-	-	-	-	-	-	-
NP 850746.1 *A. thalina* AGG2	-	-	-	-	-	-	-	-	-	-	-	-	-	-	-	-	-	-	-	-	-	-	-	-	-
*Pa*GG1	-	-	-	-	-	-	-	-	-	-	-	-	-	-	-	-	-	-	-	-	-	-	-	-	-
ADE76615.1 *P. sitchensis*	-	-	-	-	-	-	-	-	-	-	-	-	-	-	-	-	-	-	-	-	-	-	-	-	-
EX389861.1 *P. glauca*	-	-	-	-	-	-	-	-	-	-	-	-	-	-	-	-	-	-	-	-	-	-	-	-	-
CO158867.1 *P. taeda*	-	-	-	-	-	-	-	-	-	-	-	-	-	-	-	-	-	-	-	-	-	-	-	-	-
BX254993.1/BX680269.1 *P. pinaster*	-	-	-	-	-	-	-	-	-	-	-	-	-	-	-	-	-	-	-	-	-	-	-	-	-
GT252854.1 *P. contorta*	-	-	-	-	-	-	-	-	-	-	-	-	-	-	-	-	-	-	-	-	-	-	-	-	-
Phvul.001G057600.1 *P. vulgaris*	-	-	-	-	-	-	-	-	-	-	-	-	-	-	-	-	-	-	-	-	-	-	-	-	-
I3SQN8 *L. japonicus*	-	-	-	-	-	-	-	-	-	-	-	-	-	-	-	-	-	-	-	-	-	-	-	-	-
*Pa*GG2	-	-	-	-	-	-	-	-	-	-	-	-	-	-	-	-	-	-	-	-	-	-	-	-	-
DR093846 *P. taeda*	-	-	-	-	-	-	-	-	-	-	-	-	-	-	-	-	-	-	-	-	-	-	-	-	-
GT123649.1 *P. sitchensis*	-	-	-	-	-	-	-	-	-	-	-	-	-	-	-	-	-	-	-	-	-	-	-	-	-
BT117176.1 *P. glauca*	-	-	-	-	-	-	-	-	-	-	-	-	-	-	-	-	-	-	-	-	-	-	-	-	-

o

ABK26455.1 *P. sitchensis*	-	-	-	-	-	-	-	-	-	-	-	-	-	-	-	-	-	-	-	-	-	-	-	-	-
*Pa*GG3	-	-	-	-	-	-	-	-	-	-	-	-	-	-	-	-	-	-	-	-	-	-	-	-	-
BT111616.1 *P. glauca*	-	-	-	-	-	-	-	-	-	-	-	-	-	-	-	-	-	-	-	-	-	-	-	-	-
DT638145.1 *P. taeda*	-	-	-	-	-	-	-	-	-	-	-	-	-	-	-	-	-	-	-	-	-	-	-	-	-
Pp1s22 182V6.1 *P. patens*	-	-	-	-	-	-	-	-	-	-	-	-	-	-	-	-	-	-	-	-	-	-	-	-	-
Pp1s39 119V6.2*P. Patens*	-	-	-	-	-	-	-	-	-	-	-	-	-	-	-	-	-	-	-	-	-	-	-	-	-
Medtr8g021170.1 *M. truncatula*	-	-	-	-	-	-	-	-	-	-	-	-	-	-	-	-	-	-	-	-	-	-	-	-	-
Phvul.003G130200.1 *P. vulgaris*	-	S	C	K	C	T	C	T	C	S	C	P	S	-	-	-	-	-	-	C	P	K	V	S	S
Medtr4g125190.1 *M. truncatula*	-	-	-	-	-	-	-	-	-	-	-	-	-	-	-	-	-	-	-	-	-	-	-	-	-
Medtr2g042200.1 *M. truncatula*	-	D	C	K	C	S	C	S	C	T	C	P	S	-	-	-	-	-	-	F	P	K	V	R	P
Carubv10002620m *C. rubella*	K	K	K	S	S	C	C	S	C	T	C	K	I	R	W	S	S	C	C	F	K	C	P	K	V
Thhalv10014548m *T. halophila*	K	K	-	S	S	C	C	S	C	N	C	K	I	R	W	S	S	C	-	F	R	C	P	K	V
XP 002874008.1 *A. lyrata* subsp. *Lyrata*	K	K	-	S	S	C	C	S	C	N	C	K	I	R	W	S	S	C	-	F	R	C	P	K	V
AT5G20635.1 *A. thaliana* AGG3	K	K	-	S	S	C	C	S	C	N	C	K	I	R	W	S	S	C	-	F	S	C	P	K	V
ADE77369.1 *P. sitchensis*	-	-	-	-	-	-	-	-	-	-	-	-	-	-	-	-	-	-	-	-	-	-	-	-	-
*Pa*GG4	-	-	-	-	-	-	-	-	-	-	-	-	-	-	-	-	-	-	-	-	-	-	-	-	-
Phvul.007G175700.1 *P. vulgaris*	-	-	-	-	-	-	-	-	-	-	-	-	-	-	-	-	-	-	-	-	-	-	-	-	-
G7ICP9 *M. truncatula*	-	-	-	-	-	-	-	-	-	-	-	-	-	-	-	-	-	-	-	-	-	-	-	-	-
Bra007741 *B. rapa*	-	-	-	-	-	-	-	-	-	-	-	-	-	-	-	-	-	-	-	-	-	-	-	-	-
EE552948.1 *B. napus*	-	-	-	-	-	-	-	-	-	-	-	-	-	-	-	-	-	-	-	-	-	-	-	-	-
FD578839.1 *R. sativus*	-	-	-	-	-	-	-	-	-	-	-	-	-	-	-	-	-	-	-	-	-	-	-	-	-
Thhalv10006334m *T. halophila*	-	-	-	-	-	-	-	-	-	-	-	-	-	-	-	-	-	-	-	-	-	-	-	-	-
Carubv10018305m *C. rubella*	-	-	-	-	-	-	-	-	-	-	-	-	-	-	-	-	-	-	-	-	-	-	-	-	-
XP 002876721.1 *A. lyrata* subsp. *Lyrata*	-	-	-	-	-	-	-	-	-	-	-	-	-	-	-	-	-	-	-	-	-	-	-	-	-
NP 567147.1 *A. thaliana* AGG1	-	-	-	-	-	-	-	-	-	-	-	-	-	-	-	-	-	-	-	-	-	-	-	-	-
FF393368.1 *V. unguiculata*	-	-	-	-	-	-	-	-	-	-	-	-	-	-	-	-	-	-	-	-	-	-	-	-	-
XP 002883385.1 *A. lyrata* subsp. *Lyrata*	-	-	-	-	-	-	-	-	-	-	-	-	-	-	-	-	-	-	-	-	-	-	-	-	-
I3SXY5 *L. japonicus*	-	-	-	-	-	-	-	-	-	-	-	-	-	-	-	-	-	-	-	-	-	-	-	-	-
Bra023782 *B. rapa*	-	-	-	-	-	-	-	-	-	-	-	-	-	-	-	-	-	-	-	-	-	-	-	-	-
ACX43275.1 *B. napus*	-	-	-	-	-	-	-	-	-	-	-	-	-	-	-	-	-	-	-	-	-	-	-	-	-
Thhalv10021823m *T. halophila*	-	-	-	-	-	-	-	-	-	-	-	-	-	-	-	-	-	-	-	-	-	-	-	-	-
Phvul.007G111000.1 *P. vulgaris*	-	-	-	-	-	-	-	-	-	-	-	-	-	-	-	-	-	-	-	-	-	-	-	-	-
FF545146.1 *V. unguiculata*	-	-	-	-	-	-	-	-	-	-	-	-	-	-	-	-	-	-	-	-	-	-	-	-	-
I3T376 *L. japonicus*	-	-	-	-	-	-	-	-	-	-	-	-	-	-	-	-	-	-	-	-	-	-	-	-	-
Carubv10014985m *C. rubella*	-	-	-	-	-	-	-	-	-	-	-	-	-	-	-	-	-	-	-	-	-	-	-	-	-
NP 850746.1 *A. thalina* AGG2	-	-	-	-	-	-	-	-	-	-	-	-	-	-	-	-	-	-	-	-	-	-	-	-	-
*Pa*GG1	-	-	-	-	-	-	-	-	-	-	-	-	-	-	-	-	-	-	-	-	-	-	-	-	-
ADE76615.1 *P. sitchensis*	-	-	-	-	-	-	-	-	-	-	-	-	-	-	-	-	-	-	-	-	-	-	-	-	-
EX389861.1 *P. glauca*	-	-	-	-	-	-	-	-	-	-	-	-	-	-	-	-	-	-	-	-	-	-	-	-	-
CO158867.1 *P. taeda*	-	-	-	-	-	-	-	-	-	-	-	-	-	-	-	-	-	-	-	-	-	-	-	-	-
BX254993.1/BX680269.1 *P. pinaster*	-	-	-	-	-	-	-	-	-	-	-	-	-	-	-	-	-	-	-	-	-	-	-	-	-
GT252854.1 *P. contorta*	-	-	-	-	-	-	-	-	-	-	-	-	-	-	-	-	-	-	-	-	-	-	-	-	-
Phvul.001G057600.1 *P. vulgaris*	-	-	-	-	-	-	-	-	-	-	-	-	-	-	-	-	-	-	-	-	-	-	-	-	-
I3SQN8 *L. japonicus*	-	-	-	-	-	-	-	-	-	-	-	-	-	-	-	-	-	-	-	-	-	-	-	-	-
*Pa*GG2	-	-	-	-	-	-	-	-	-	-	-	-	-	-	-	-	-	-	-	-	-	-	-	-	-
DR093846 *P. taeda*	-	-	-	-	-	-	-	-	-	-	-	-	-	-	-	-	-	-	-	-	-	-	-	-	-
GT123649.1 *P. sitchensis*	-	-	-	-	-	-	-	-	-	-	-	-	-	-	-	-	-	-	-	-	-	-	-	-	-
BT117176.1 *P. glauca*	-	-	-	-	-	-	-	-	-	-	-	-	-	-	-	-	-	-	-	-	-	-	-	-	-

p

ABK26455.1 *P. sitchensis*	-	-	-	-	-	-	-	-	-	-	-	-	-	-	-	-	-	-	-	-	-	-	-	-	-
*Pa*GG3	-	-	-	-	-	-	-	-	-	-	-	-	-	-	-	-	-	-	-	-	-	-	-	-	-
BT111616.1 *P. glauca*	-	-	-	-	-	-	-	-	-	-	-	-	-	-	-	-	-	-	-	-	-	-	-	-	-
DT638145.1 *P. taeda*	-	-	-	-	-	-	-	-	-	-	-	-	-	-	-	-	-	-	-	-	-	-	-	-	-
Pp1s22 182V6.1 *P. patens*	-	-	-	-	-	-	-	-	-	-	-	-	-	-	-	-	-	-	-	-	-	-	-	-	-
Pp1s39 119V6.2*P. Patens*	-	-	-	-	-	-	-	-	-	-	-	-	-	-	-	-	-	-	-	-	-	-	-	-	-
Medtr8g021170.1 *M. truncatula*	-	-	-	-	-	-	-	-	-	-	-	-	-	-	-	-	-	-	-	-	-	-	-	-	-
Phvul.003G130200.1 *P. vulgaris*	C	C	C	C	-	-	-	-	T	K	S	-	-	-	C	W	R	P	C	-	C	L	C	C	-
Medtr4g125190.1 *M. truncatula*	-	-	-	-	-	-	-	-	-	-	-	-	-	-	-	-	-	-	-	-	-	-	-	-	-
Medtr2g042200.1 *M. truncatula*	C	G	S	C	K	S	Y	-	S	Y	L	-	-	D	K	P	N	K	Q	L	C	L	A	I	I
Carubv10002620m *C. rubella*	R	L	S	S	C	C	F	-	C	N	C	K	N	P	C	S	N	P	C	-	C	L	A	F	-
Thhalv10014548m *T. halophila*	R	L	C	-	C	C	F	-	C	N	C	K	N	L	C	S	N	P	C	-	C	L	A	F	-
XP 002874008.1 *A. lyrata* subsp. *Lyrata*	R	L	C	S	C	C	F	-	C	N	C	K	N	L	C	S	N	P	C	-	C	L	A	F	-
AT5G20635.1 *A. thaliana* AGG3	R	L	C	S	C	C	F	-	C	N	C	K	N	L	C	S	N	P	C	-	C	L	A	F	-
ADE77369.1 *P. sitchensis*	-	-	-	-	-	-	-	-	-	-	-	-	-	-	-	-	-	-	-	-	-	-	-	-	-
*Pa*GG4	-	-	-	-	-	-	-	-	-	-	-	-	-	-	-	-	-	-	-	-	-	-	-	-	-
Phvul.007G175700.1 *P. vulgaris*	-	-	-	-	-	-	-	-	-	-	-	-	-	-	-	-	-	-	-	-	-	-	-	-	-
G7ICP9 *M. truncatula*	-	-	-	-	-	-	-	-	-	-	-	-	-	-	-	-	-	-	-	-	-	-	-	-	-
Bra007741 *B. rapa*	-	-	-	-	-	-	-	-	-	-	-	-	-	-	-	-	-	-	-	-	-	-	-	-	-
EE552948.1 *B. napus*	-	-	-	-	-	-	-	-	-	-	-	-	-	-	-	-	-	-	-	-	-	-	-	-	-
FD578839.1 *R. sativus*	-	-	-	-	-	-	-	-	-	-	-	-	-	-	-	-	-	-	-	-	-	-	-	-	-
Thhalv10006334m *T. halophila*	-	-	-	-	-	-	-	-	-	-	-	-	-	-	-	-	-	-	-	-	-	-	-	-	-
Carubv10018305m *C. rubella*	-	-	-	-	-	-	-	-	-	-	-	-	-	-	-	-	-	-	-	-	-	-	-	-	-
XP 002876721.1 *A. lyrata* subsp. *Lyrata*	-	-	-	-	-	-	-	-	-	-	-	-	-	-	-	-	-	-	-	-	-	-	-	-	-
NP 567147.1 *A. thaliana* AGG1	-	-	-	-	-	-	-	-	-	-	-	-	-	-	-	-	-	-	-	-	-	-	-	-	-
FF393368.1 *V. unguiculata*	-	-	-	-	-	-	-	-	-	-	-	-	-	-	-	-	-	-	-	-	-	-	-	-	-
XP 002883385.1 *A. lyrata* subsp. *Lyrata*	-	-	-	-	-	-	-	-	-	-	-	-	-	-	-	-	-	-	-	-	-	-	-	-	-
I3SXY5 *L. japonicus*	-	-	-	-	-	-	-	-	-	-	-	-	-	-	-	-	-	-	-	-	-	-	-	-	-
Bra023782 *B. rapa*	-	-	-	-	-	-	-	-	-	-	-	-	-	-	-	-	-	-	-	-	-	-	-	-	-
ACX43275.1 *B. napus*	-	-	-	-	-	-	-	-	-	-	-	-	-	-	-	-	-	-	-	-	-	-	-	-	-
Thhalv10021823m *T. halophila*	-	-	-	-	-	-	-	-	-	-	-	-	-	-	-	-	-	-	-	-	-	-	-	-	-
Phvul.007G111000.1 *P. vulgaris*	-	-	-	-	-	-	-	-	-	-	-	-	-	-	-	-	-	-	-	-	-	-	-	-	-
FF545146.1 *V. unguiculata*	-	-	-	-	-	-	-	-	-	-	-	-	-	-	-	-	-	-	-	-	-	-	-	-	-
I3T376 *L. japonicus*	-	-	-	-	-	-	-	-	-	-	-	-	-	-	-	-	-	-	-	-	-	-	-	-	-
Carubv10014985m *C. rubella*	-	-	-	-	-	-	-	-	-	-	-	-	-	-	-	-	-	-	-	-	-	-	-	-	-
NP 850746.1 *A. thalina* AGG2	-	-	-	-	-	-	-	-	-	-	-	-	-	-	-	-	-	-	-	-	-	-	-	-	-
*Pa*GG1	-	-	-	-	-	-	-	-	-	-	-	-	-	-	-	-	-	-	-	-	-	-	-	-	-
ADE76615.1 *P. sitchensis*	-	-	-	-	-	-	-	-	-	-	-	-	-	-	-	-	-	-	-	-	-	-	-	-	-
EX389861.1 *P. glauca*	-	-	-	-	-	-	-	-	-	-	-	-	-	-	-	-	-	-	-	-	-	-	-	-	-
CO158867.1 *P. taeda*	-	-	-	-	-	-	-	-	-	-	-	-	-	-	-	-	-	-	-	-	-	-	-	-	-
BX254993.1/BX680269.1 *P. pinaster*	-	-	-	-	-	-	-	-	-	-	-	-	-	-	-	-	-	-	-	-	-	-	-	-	-
GT252854.1 *P. contorta*	-	-	-	-	-	-	-	-	-	-	-	-	-	-	-	-	-	-	-	-	-	-	-	-	-
Phvul.001G057600.1 *P. vulgaris*	-	-	-	-	-	-	-	-	-	-	-	-	-	-	-	-	-	-	-	-	-	-	-	-	-
I3SQN8 *L. japonicus*	-	-	-	-	-	-	-	-	-	-	-	-	-	-	-	-	-	-	-	-	-	-	-	-	-
*Pa*GG2	-	-	-	-	-	-	-	-	-	-	-	-	-	-	-	-	-	-	-	-	-	-	-	-	-
DR093846 *P. taeda*	-	-	-	-	-	-	-	-	-	-	-	-	-	-	-	-	-	-	-	-	-	-	-	-	-
GT123649.1 *P. sitchensis*	-	-	-	-	-	-	-	-	-	-	-	-	-	-	-	-	-	-	-	-	-	-	-	-	-
BT117176.1 *P. glauca*	-	-	-	-	-	-	-	-	-	-	-	-	-	-	-	-	-	-	-	-	-	-	-	-	-
