# Supplementary material for: Heterotrimeric G–proteins in Picea abies and their regulation in response to Heterobasidion annosum s.l. infection
Source: BMC Plant Biol. 2015 Dec 12;15:287. doi: 10.1186/s12870-015-0676-1 (PMC4676809; doi:10.1186/s12870-015-0676-1)
Supplement: Additional file 8: — Primer sequences for full-length amplification of the Gα-, Gβ- and Gγ-subunits cloning of the sequences and qPCR. (DOCX 17 kb) [file 12870_2015_676_MOESM8_ESM.docx]

Additional file 9 Primer sequences for full-length amplification of the Gα-, Gβ and G**γ**-subunits, cloning of the sequences and qPCR

| **Primer name** | **Forward sequence** | **Reverse sequence** | **Annealing temperature** | **use** |
| --- | --- | --- | --- | --- |
| G alpha 1 | 5’-AAGCTTTTCTTGAAATAGTGTACCG 3’ | 5’ TCTGCACAGACCATTTGCAC 3’ |  | Cloning of PaGPA1 |
| BetaFL | 5’ CTATGTCGGTGGCAGACCT 3’ | 5’ TCACCAATCAAGCTATTCTTC 3’ |  | Nested PCR; cloning |
| MEEET1/GG1 | 5’ CATGGAGGAGGAAACAGACA 3’ | 5’ TGTCCACGCCAACTGATTAC 3’ |  | Nested 5’ RACE; cloning of GG1 |
| MINKS 1 F/GG3 F | 5’ ACCCAGAGTGCAGATTCGTT 3’ | - |  | Nested 3’ RACE; cloning of GG3 |
| MQGT 1 F/GG2 F | 5’ CTAAGCATTTGATGGGCAAA 3’ | - |  | Nested 3’ RACE; cloning of GG2 |
| MIK F outer/GG4 F outer | 5’ AAAACCAGCAAACCCAAATG 3’ | - |  | Nested 3’ RACE; cloning of GG4 |
| MIK F inner/GG4 F inner | 5’ AAACCCAAATGGAGGAGTGT 3’ | - |  | Nested 3’ RACE; cloning of GG4 |
| Universal Primer Mix (UPM) (1) | - | Long:  5’  CTAATACGACTCACTATAGGGCAAGCA  GTGGTATCAACGCAGAGT 3’  Short:  5’ CTAATACGACTCACTATAGGGC 3’ |  | Nested 3’ RACE; cloning of GG2, GG3 and GG4 |
| Nested Universal Primers (NUP) (1) | - | 5’ AAGCAGTGGTATCAACGCAGAGT 3’  control reagents |  | Nested 3’ RACE; cloning of GG2, GG3 and GG4 |
| M13 (2) | 5´ GTAAAACGACGGCCAG 3´ | 5’ CAGGAAACAGCTATGAC 3‘ | 55°C | M13 screening |
| ELF1 alpha spruce (3) | 5’ TGGCAAGGAACTGGAGAAGGAA 3’ | 5’ TAGTCCCTCACAGCAAAACGA 3’ | 60°C | qPCR |
| ELF4 alpha spruce (4) | 5’ AGTAAGCCCGTGAGGATTC 3’ | 5’ AGTCAGCCAGTCAACCTTTC 3’ | 60°C | qPCR |
| PG mutase (5) | 5’ AATGCAGTTGAAGCCATTCC 3’ | 5’ CCAGTGCCGAAACTCTCTTTC 3’ | 60°C | qPCR |
| G alpha | 5’ CCTGCTGACCGTGTCGATA 3’ | 5’ CTCAGAGTGAGAGTTTCATC 3’ | 60°C for spruce/ 58°C for pine | qPCR |
| G beta | 5’ GCTGTCTTGGATTGTCAGCT 3’ | 5’ CTTCGGTGGCCTCCAAATG 3’ | 60°C | qPCR |
| MEEET/GG1 | 5’ TACAGGAGCTCAAAATGGTC 3 | 5’ CACCCACATCCGTCTCTGT 3’ | 60°C for spruce/ 58°C for pine | qPCR GG1 |
| MQGT/GG2 | 5’ CCAGCACTATAGGTCCAGA 3’ | 5’ TCATAGACAACATCCACAGC 3’ | 60°C | qPCR GG2 |
| MINKS/GG3 | 5’ TATCATCCTGGGATCGATGG 3’ | 5’ CACATGCTTACAAGAACAGC 3’ | 60°C for spruce/ 58°C for pine | qPCR GG3 |
|  |  |  |  |  |

(1) UMP and NUP primers are supplied with the Clontech SMARTer™ RACE cDNA Amplification Kit; (2) M13 primers are supplied by the Invitrogen TOPO TA Cloning pCR®2.1 kit; (3) Arnerup et al. 2011; (4) Palovaara and Hakman 2008; (5) Vestman et al. 2010
